# The impact of cross-docked poses on performance of machine learning classifier for protein–ligand binding pose prediction

**DOI:** 10.1186/s13321-021-00560-w

**Published:** 2021-10-16

**Authors:** Chao Shen, Xueping Hu, Junbo Gao, Xujun Zhang, Haiyang Zhong, Zhe Wang, Lei Xu, Yu Kang, Dongsheng Cao, Tingjun Hou

**Affiliations:** 1 Innovation Institute for Artificial Intelligence in Medicine of Zhejiang University, College of Pharmaceutical Sciences, Zhejiang University, Hangzhou, Zhejiang 310058 People’s Republic of China; 2 State Key Lab of CAD&CG, Zhejiang University, Hangzhou, Zhejiang 310058 People’s Republic of China; 3 Xiangya School of Pharmaceutical Sciences, Central South University, Changsha, Hunan 410013 People’s Republic of China; 4 Institute of Bioinformatics and Medical Engineering, School of Electrical and Information Engineering, Jiangsu University of Technology, Changzhou, 213001 China

**Keywords:** Scoring function (SF), Machine learning (ML), Molecular docking, Protein–ligand binding pose, Cross-docking

## Abstract

Structure-based drug design depends on the detailed knowledge of the three-dimensional (3D) structures of protein–ligand binding complexes, but accurate prediction of ligand-binding poses is still a major challenge for molecular docking due to deficiency of scoring functions (SFs) and ignorance of protein flexibility upon ligand binding. In this study, based on a cross-docking dataset dedicatedly constructed from the PDBbind database, we developed several XGBoost-trained classifiers to discriminate the near-native binding poses from decoys, and systematically assessed their performance with/without the involvement of the cross-docked poses in the training/test sets. The calculation results illustrate that using Extended Connectivity Interaction Features (ECIF), Vina energy terms and docking pose ranks as the features can achieve the best performance, according to the validation through the random splitting or refined-core splitting and the testing on the re-docked or cross-docked poses. Besides, it is found that, despite the significant decrease of the performance for the threefold clustered cross-validation, the inclusion of the Vina energy terms can effectively ensure the lower limit of the performance of the models and thus improve their generalization capability. Furthermore, our calculation results also highlight the importance of the incorporation of the cross-docked poses into the training of the SFs with wide application domain and high robustness for binding pose prediction. The source code and the newly-developed cross-docking datasets can be freely available at https://github.com/sc8668/ml_pose_prediction and https://zenodo.org/record/5525936, respectively, under an open-source license. We believe that our study may provide valuable guidance for the development and assessment of new machine learning-based SFs (MLSFs) for the predictions of protein–ligand binding poses.

**Supplementary Information:**

The online version contains supplementary material available at 10.1186/s13321-021-00560-w.

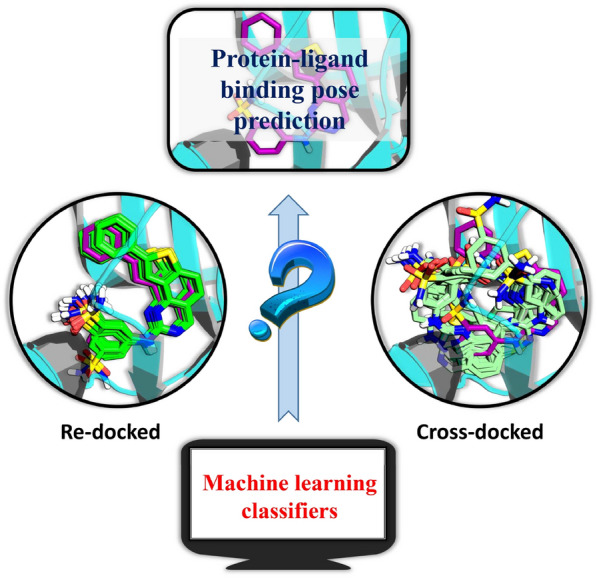

## Introduction

Molecular docking is one of the core technologies in structure-based drug design (SBDD), and it has contributed enormously to drug discovery and development in the past decades [1–3]. Typically, molecular docking has two stages: (1) sampling the pose of the ligand in the binding site of a macromolecular target (usually a protein) and (2) scoring the binding strength of the ligand to the target by using a predefined scoring function (SF). Despite impressive success of molecular docking, the deficiency of SFs remains a major obstacle to the reliability of real-world applications of docking [4, 5].

Depending on different theoretical principles, existing SFs can be typically divided into four main groups: physics-based, empirical, knowledge-based, and newly-emerged machine learning-based SFs (MLSFs) [6]. The former three can be collectively referred to as classical SFs, since all of them follow an additive formulated hypothesis to represent the relationship between the features that characterize protein–ligand interactions and experimental bioactivities. With the aid of state-of-the-art machine learning (ML) algorithms, MLSFs developed by automatically learning the generalized nonlinear functional forms from the training data have gradually emerged as a promising alternative to overcome the disadvantages of classical SFs. During the past few years, extensive efforts have been made towards the development of MLSFs, ranging from traditional ML-based approaches (e.g., RF-Score [7–9], NNScore [10, 11], MIEC-SVM [12], Δ_Vina_RF_20_ [13], and AGL-Score [14]) to recently-emerged deep learning (DL)-based methods (e.g., AtomNet [15], DeepVS [16], K_DEEP_ [17], and PotentialNet [18]), and most of these MLSFs demonstrate remarkably superior performance compared with the classical SFs [19–22].

Typically, three major metrics are used to evaluate the performance of a certain SF, i.e., the ability to produce binding scores that linearly correlate with experimentally determined affinities (scoring power), the ability to discriminate near-native ligand binding pose from decoys (docking power), and the ability to identify active compounds from decoys (screening power). Classical SFs are usually constructed based on the datasets where the crystal structure and the binding affinity for each protein–ligand complex have been experimentally determined (e.g., PDBbind [23]), and then they can be generalized to either binding pose prediction or structure-based virtual screening (SBVS). However, that is not the case for MLSFs. Though most MLSFs built in a similar way exhibited better scoring power than classical SFs, their docking power and screening power are usually unsatisfactory, implying that the generalization capability of MLSFs may be still questionable [24–27]. Thus, building different MLSFs for specific tasks (i.e., binding pose prediction, binding affinity prediction or virtual screening) with the involvement of decoy poses and/or inactive compounds in the training set is a mainstream strategy rather than building a single generalized MLSF. Recently, the scoring power and screening power of a number of MLSFs have been systematically assessed [20–22, 27–35], and in this study we tend to investigate the capability of MLSFs in binding pose prediction.

Accurate identification of near-native binding poses from decoy poses is a prerequisite for many downstream simulation tasks, such as binding affinity prediction and SBVS. Over the last few years, a number of MLSFs for binding pose prediction have been reported [26, 36–46]. Some of them were trained to explicitly predict the root-mean-square-deviation (RMSD) values of binding poses, while the others were trained to directly distinguish near-native poses from high-RMSD ones. In 2015, Ashtawy and Mahapatra first developed MLSFs for the prediction of RMSD values, and the top RMSD-based SF can yield a success rate of ~ 80%, significantly higher than 70% of the best empirical SF [36]. They also found that the RMSD-based method can provide more than 120% improvement on docking task over the counterparts trained for binding affinity prediction [38]. In 2017, Ragoza et al. implemented their three-dimension (3D) grid-based convolutional neural network (CNN) architecture to build a ML classifier for pose prediction [37]. They found that the method performed consistently well in an inter-target pose prediction test, but it could hardly beat the classical Autodock Vina in an intra-target pose ranking test, which we are more concerned about. In 2020, Morrone et al. proposed a dual-graph neural network model for pose prediction, which was concatenated by a ligand-only sub-graph to store ligand structures and an interaction-based sub-graph to represent protein–ligand interaction information [40]. Similarly, this model outperformed Autodock Vina in terms of the area under the receiver operating characteristic curve (AUROC) but did not show improved performance regarding the top 1 success rate. Then, they incorporated the docking pose ranks into training as an additional feature, and the retrained model showed better performance than Vina. Despite so, a common defect of the above studies is that they can only be compared with themselves or with the classical SFs but can hardly be compared to each other because the building procedures for these models are quite different, such as different dataset partitioning methods and different pose generation (docking) methods. Besides, in most cases only the re-docked poses (re-docking the co-crystalized ligands into the pockets) were used in model training/testing, but actually, re-docking is just an artificial exercise, which completely neglects the induced fit or conformational change of the targets that occur upon ligand binding. This perfect match between protein and ligand can be hardly obtained in a real-world prospective exercise. Very recently, Francoeur et al. reported a standardized dataset named CrossDocked2020 set with 22.5 million poses generated by docking ligands into multiple similar binding pockets to better mimic the real-world scenarios, and they comprehensively estimated the scoring and docking powers of their grid-based CNN models [42]. Based on the dataset and assessment results, they further released the 1.0 version of GNINA, which could be considered as the first publicly available docking software that integrated an ensemble of CNNs as a SF [46]. However, it seems that the dataset may be not so suitable for the large-scale assessment of SFs due to its complexity and randomness. Moreover, many recent publications put more focus on DL-based models, but some traditional ML-based approaches that exhibit comparative or even better performance in many cases may also deserve attention [47–49].

In this study, two sets of descriptors that had been well validated in binding affinity prediction tasks, including the NNscore features [10, 11] and Extended Connectivity Interaction Features (ECIF) [47], were used to build the MLSFs for binding pose prediction utilizing the extreme gradient boosting (XGBoost) algorithm. In addition, the impacts of the incorporation of classical energy terms and docking pose ranks as the features on the performance of MLSFs were explored. The MLSFs were dedicatedly validated through three validation strategies, including random splitting, refined-core splitting, and threefold clustered-cross-validation. Besides the routine investigation based on the re-docked poses from PDBbind, several PDBbind-based datasets for cross-docking tests (e.g. PDBbind-CrossDocked-Core and PDBbind-CrossDocked-Refined) were constructed to investigate some important aspects of MLSFs, including their sensitivity to crystal structures, their sensitivity to docking programs, and the impacts of re-docked and cross-docked poses on each other.

## Methods

### Dataset construction and preparation

The PDBbind [23, 50] database (http://www.pdbbind.org.cn/) that contains a consolidated repository of the binding affinity data for a wide range of biomolecular complexes deposited in the Protein Data Bank (PDB) [51] serves as a core dataset for the development and benchmarking of SFs. The refined set of PDBbind2016 that had been dedicatedly examined and prepared in our previous study [30] was employed as the base dataset here. The detailed information of each dataset utilized in this study is summarized in Table 1.

**Table 1:** The information of the datasets utilized in this study

Dataset	Re-docked poses				Cross-docked poses			
	Complexes	Poses	Positives	Negatives	Complexes	Poses	Positives	Negatives
PDBbind-ReDocked	4057	83,876	39,978	43,898	–	–	–	–
PDBbind-ReDocked-Refined	3767	77,922	37,114	40,808	–	–	–	–
PDBbind-ReDocked-Core	290	5954 (5664)^a^	2864 (2574)	3090	–	–	–	–
CASF-Docking	285^b^	22,777 (22,492)	5494 (5209)	17,283	–	–	–	–
PDBbind-CrossDocked-Core-s	285	5551	2565	2,986	1058	20,859	5872	14,987
PDBbind-CrossDocked-Core-g	282	4795	1596	3,199	1030	17,814	3768	14,046
PDBbind-CrossDocked-Core-v	285	5693	301	5,392	1058	21,145	740	20,405
PDBbind-CrossDocked-Refined	3767	77,839 (74,072)	37,028 (33,261)	40,811	90,002	1,874,433	1,499,702	374,731
PDBbind-CrossDocked-Refined*^c^	3767	77,839 (74,072)	37,028 (33,261)	40,811	90,002	1,731,351	1,428,161	303,190

^a^The number in bracket refers to the number after removing the crystal poses

^b^The core set of original PDBbind 2016 has 290 complexes belonging to 58 clusters, while only 285 are remained when constructing the CASF because there is a duplicated cluster

^c^The set eliminates the cross-native poses

#### PDBbind-ReDocked

Previous studies were accustomed to using AutoDock Vina/Smina [52, 53] to generate docking poses due to its free of charge and acceptable accuracy, but here we used Surflex-Dock [54], one of the best-performing docking programs in our previous assessments [55, 56] to reproduce the native binding pose when the best pose with the lowest RMSD among the top 20 scoring poses was utilized as the final pose. The docking was conducted with the ‘-pgeom’ mode, and up to 20 poses were generated for each ligand. To guarantee that each complex in the training set has at least one low-RMSD pose, the crystal poses were also mixed into the dataset, thus resulting in a total of 4057 complexes and 83,876 poses. The heavy-atom RMSD between each docking pose and the crystal pose was calculated using the *obrms* utility implemented in OpenBabel [57], and the poses with RMSD less than 2.0 Å were considered as near-native. Finally, the dataset (https://zenodo.org/record/5525936/files/PDBbind-CrossDocked-Core.tar.bz2) contains 39,978 positives and 43,898 negatives.

#### CASF-Docking

Comparative Assessment of Scoring Functions (CASF) [58] benchmark (http://www.pdbbind.org.cn/casf.php) based on a subset of PDBbind (core set) can be considered as a golden standard for the assessment of classical SFs, and it contains four subsets to assess the four aspects of a SF. The subset to assess docking power (CASF-Docking) contains 285 protein–ligand complexes, and ∼1000 docking poses was generated for each complex using three popular docking programs (GOLD, Surflex-Dock, and MOE Dock) to achieve the maximal conformational diversity. Finally, up to 100 poses was selected by clustering for each complex, thus generating a total of 22,777 poses (5494 positives and 17,283 negatives). The details of the pose generation process can be found in Ref [58]. This dataset serves as an external test set. It should be noted that a fairly complete coverage of the possible binding poses is provided in this dataset because multiple docking programs were utilized and a further clustering operation was conducted.

#### PDBbind-CrossDocked-Core

The 285 protein–ligand complexes in the core set of PDBbind were clustered into 57 groups by protein sequence similarity with 5 complexes in each cluster. Therefore, the complexes within each cluster were aligned using the *structalign* utility in Schrödinger [59], and then cross docking was carried out by docking a certain ligand in a crystal structure into the pockets of the other four crystal structures in the same cluster. In order to explore the sensitivity of MLSFs to different docking programs, besides Surflex-Dock, Glide SP [60] and AutoDock Vina were also used to generate the binding poses. For Glide SP, the receptor grids centered on the co-crystallized ligand were defined with the size of the binding box of 10 × 10 × 10 Å. For AutoDock Vina, the size of the search space was set to 30 × 30 × 30 Å, and the maximum energy difference between the best and the worst binding modes was set to 100 kcal/mol. For both programs, up to 20 poses were generated, and the other parameters were set to default. Meanwhile, the docking results were visually inspected to guarantee that the cross docking was just conducted for the complexes with the ligands in the same pockets and without residue mutations in the pockets. Of course, the complexes failing in docking were removed. The three datasets (https://zenodo.org/record/5525936/files/PDBbind-CrossDocked-Core.tar.bz2) generated by Surflex-Dock (PDBbind-CrossDocked-Core-s), Glide SP (PDBbind-CrossDocked-Core-g) and Vina (PDBbind-CrossDocked-Core-v) contain 1343 complexes and 26,410 poses (8437 positives and 17,973 negatives), 1312 complexes and 22,609 poses (5364 positives and 17,245 negatives), and 1343 complexes and 26,838 poses (1041 positives and 25,797 negatives), respectively. It should be noted that PDBbind-CrossDocked-Core-v is an extremely difficult set because most poses are marked as the negatives. This may be mainly caused by the default post-docking operations in Vina, which clusters the resulting poses using a relatively high RMSD cutoff, and therefore only a few near-native poses are finally obtained.

#### PDBbind-CrossDocked-Refined

Based on the Uniprot IDs provided in PDBbind, the refined set excluding the complexes in the core set can be divided into 1302 clusters (in which 749 clusters have only one complex). The re-docking operation is the same as that for PDBbind-ReDocked, while for the clusters with more than one complex, cross docking was carried out by Surflex-Dock, thus generating a mixed dataset composed of both the re-docked and cross-docked poses (PDBbind-CrossDocked-Refined, https://zenodo.org/record/5525936/files/PDBbind-CrossDocked-Refined.tar.bz2), which contains 93,769 complexes and 1,964,686 poses.

### Feature calculation

Two sets of descriptors that had been well validated [10, 11, 30, 47], i.e., the NNscore features and Extended Connectivity Interaction Features (ECIF), were tested in this study, and the simple element atom-type pairwise counts (ELEM) and extended three-dimensional fingerprint (E3FP) [61] were utilized for comparison. Besides, we also tried to incorporate the Vina energy terms and docking pose ranks into the training of MLSFs.

#### NNscore

NNscore proposed by Durrant et al. is a pioneer MLSF [10], and the MLSF reported by our study based on the NNscore descriptors show excellent performance to binding affinity prediction [30]. A total of 348 descriptors are used by the second version of NNscore [11], and they can encode the interaction pattern for a protein–ligand complex from multiple aspects. The five energy terms used by NNscore were directly computed by AutoDock Vina, and the other features were calculated by BINANA [62], which provide 12 different binding characteristics ranging from the number of hydrogen bonds to rough metrics of active-site flexibility.

#### ECIF and ELEM

ELEM is a set of simple protein–ligand atom-type pairwise counts, which was first used by RF-Score [7]. Here four types of protein atoms (C, N, O, and S) and nine types of ligand atoms (C, N, O, S, P, F, Cl, Br, and I) within 6.0 Å around the pockets were considered, and then a total of 36 features could be computed. ECIF is also a set of atom-type pairwise counts but takes each atom’s connectivity into account. A total of 22 protein atom types and 71 ligand atom types, defined by atomic element, the number of explicit valences, the number of attached heavy atoms, the number of attached hydrogen atoms, whether is aromatic and whether is in a ring, are used to characterize each atom, resulting in a total of 1562 features. These two types of descriptors were calculated by the scripts based on the RDKit toolkit (version 2019.03.1) [63].

##### E3FP

The E3FP [61] fingerprints are developed based on the logic of the extended connectivity fingerprints (ECFP) [64], a class of widely-used topological fingerprints based on the Morgan algorithm. Given a specific conformer, E3FP can generate a 3D fingerprint parameterized by a shell radius multiplier *r* and the maximum number of iterations *L*. Here, all the fingerprints were generated based on the docking poses with the default settings, and the hashed fingerprints with 1024 bits were ultimately generated. For this set of features, only the 3D conformations of ligands are needed, thus serving as a reference to judge whether our MLSFs could consistently learn the interaction information.

### Validation methods

For the re-docked experiments, three validation methods, i.e., random splitting, refined-core splitting, and threefold clustered-cross validation (CCV), were employed, while for the cross-docked experiments, only the refined-core splitting approach was utilized. It should be noted that all the data was partitioned based on the targets rather than the poses.

#### Random splitting

This validation approach can best mimic the real-world scenarios because we can hardly judge whether the tested sample is novel enough. Here the whole refined set (4057 complexes) was randomly split into the training and test sets with the ratio of 4:1, and the whole operation was repeated by 10 times to yield a more convincing result.

#### Refined-core splitting

The core set of PDBbind has been widely used for the evaluation of SFs, and the goal of the refined-core splitting is to have a better comparison between our SFs and the methods reported by other studies. Here the core set (290 complexes) was used as the test set and the remaining (3767 complexes) were used as the training set.

#### threefold clustered-cross validation (CCV)

The aforementioned two validation methods may yield over-optimistic performance because some complexes between the training and test sets have high protein/ligand structural similarity. Hence CCV was employed to roughly estimate the generalization capability of the constructed models. The whole dataset was equally clustered into three subsets, where the proteins in different sets should have low sequence similarity and at the same time the ligands should have low structural similarity. The ligand similarity was determined by the Tanimoto similarity based on the RDKit topological fingerprints, while the sequence similarity was measured by computing the pairwise distance matrix using the *pairwise2.align.globalxx* module implemented in biopython [65]. The similarity thresholds for the ligands and proteins were set to 0.9 and 0.5, respectively, while 0.3 for the proteins if the cognate ligands were similar. As shown in Additional file 1: Figure S1, the samples in different subsets indeed satisfy the requirements of low sequence similarity and low ligand structural similarity. Any two sets were used as the training set and the other one as the test set, and the training and testing process was repeated 3 times. The whole operation was carried out using the script modified from *clustering.py* provided by Francoeur et al. [42].

### Model construction

The features with the variance less than 0.01 were removed, followed by the standardization of the remaining features using the *sklearn.preprocessing* [66] module. Extreme gradient boosting (XGBoost) [67], a well-validated ML algorithm that has been widely used in the field of computer-aided drug design (CADD) [28, 29, 31], was utilized to construct the classification models. Some major hyper-parameters (Additional file 1: Table S1) were tuned with the *hyeropt* [68] package and determined by the AUROC statistic based on the fivefold cross-validation. The maximum iteration was set to 60 with Tree Parzen estimator as the optimization algorithm. Along with the best hyper-parameter combination, the model was trained on the training set and then evaluated on the corresponding test set. XGBoost was implemented by the *xgboost* package [67]. In addition to the classifiers, we also built several regressors to directly predict the RMSD values. However, a simple experiment based on the refined-core splitting of the PDBbind-ReDocked dataset indicates that most regressors perform no better than their corresponding classifiers (Additional file 1: Table S2), and hence the classifiers were utilized for the following exploration. The seeds for *xgboost* and *hyperopt* were fixed to 2399 and 123, respectively, for others to reproduce our work.

### Baselines

Besides the ML-based approaches mentioned above, we also utilized several classical methods for comparison, including the docking scores from Surflex-Dock (or AutoDock Vina or Glide SP), the Vina scores extracted from the NNscore features, empirical SF X-Score [69] and more robust Prime-MM/GBSA [53]. For X-Score, the *FixPDB* and *FixMol2* utilities were first utilized to prepare the protein and ligand files, respectively, and then the average score of the three individual SFs available in X-Score was employed for rescoring the binding poses. Prime-MM/GBSA was executed with the *prime_mmgbsa* utility implemented in Schrödinger. The rescoring was conducted with the variable-dielectric generalized Born (VSGB) solvation model and OPLS2005 force field.

### Evaluation metrics

With the predicted probabilities/scores obtained from the ML classifiers/classical SFs, AUROC and Spearman’s rank correlation coefficient (Rs) could be calculated to evaluate the ranking capabilities of the MLSFs. The ROC curve that describes the relationship between true positive rate and false positive rate can indicate how well a model is able to distinguish low-RMSD poses from incorrect poses overall, and the corresponding AUC value ranges from 0 for a complete failure to 0.5 for a random prediction to 1 for a perfect classification, while Rs can quantitatively represent the correlation between the pose ranks predicted by each model/SF and their RMSD values. Here we defined two types of metrics, including inter-target metrics (inter-AUROC and inter-Rs) and intra-target metrics (intra-AUROC and intra-Rs). The former is computed directly based on all the tested complexes and poses, and can to some extent reflect the overall ranking capability of the models/SFs for all the groups of protein–ligand binding poses. The latter is calculated just within a specific protein–ligand complex (here at most 20 poses for a certain ligand), and then the average of all complexes is utilized to represent the final results.

The most important and intuitive metric for docking power should be the success rate (SR). For a certain complex, if one of the RMSD values of the top-ranked poses is below the predefined cutoff (usually 2.0 Å [58]), this complex can be marked as a successful prediction for the given MLSF. The analysis was performed over all the complexes in the test set, and then an overall SR was obtained by calculating the percentage of the successful cases among all the cases. For both the re-docked and cross-docked poses, the poses generated by a single docking campaign were considered as a single set, and the resulting success rate was used as the main metric. Besides, when evaluating the cross-docked poses, we also tried to consider all the docked poses of a ligand across multiple crystal structures as a single set, using the idea of ensemble docking just as Francoeur et al. did in their study [42]. As all the MLSFs developed in this study were designed for rescoring, we specifically focused on the situation when just the top 1 poses were used to calculate the success rate (SR_1_). Of course, the SR involving the top 3 poses (SR_3_) was also provided for reference.

For the random splitting and threefold CCV, the averages and standard deviations were directly obtained for analyses, while for the refined-core splitting, the random sampling of 1000 redundant copies with replacement was conducted for each statistic and the average score was calculated. In addition, we employed the Wilcoxon signed-rank test to judge whether the difference between any two compared methods was statistically significant. The difference with the P-value less than 0.05 was considered to be statistically significant.

## Results and discussion

### Comparison based on the random splitting of the re-docked poses

**Fig. 1:**
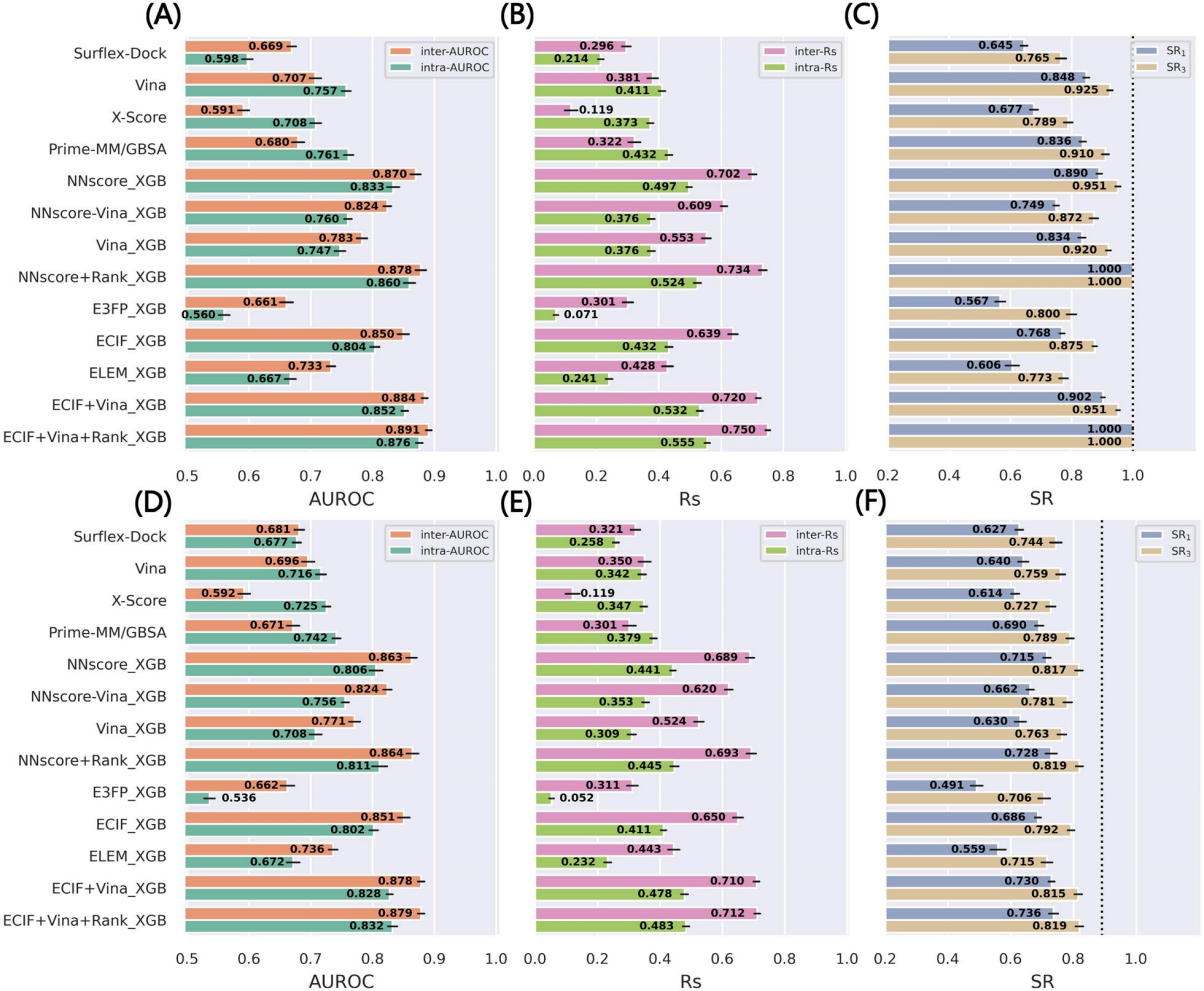
The performance in terms of **A**, **D** inter-AUROC and intra-AUROC, **B**, **E** inter-Rs and intra-Rs, and **C**, **F** top1 and top3 success rates based on the random splitting of the PDBbind-ReDocked dataset. For **A**–**C**, the crystal poses in the test set is remained while for **D**–**F** is removed. The error bars represent the standard deviation of the 10 repetitions, and the dotted lines in **C** and **F** indicate the ceiling of the success rate

The performance of our MLSFs was first evaluated by the random splitting of the PDBbind-ReDocked dataset. The comparison of Fig. 1 A–C and Fig. 1D–F indicates that whether adding the crystal poses into the test set poses a great influence on the success rates, despite minor impacts on AUC and Rs. Some models with the incorporation of the docking pose ranks can even yield SR of 1.0. This is not surprising because AUC and Rs usually reflect the overall performance for which the introduction of several easily-distinguished poses may produce almost no effects, while SR is determined by the top-ranked poses for a certain complex and hence quite sensitive to the crystal poses whose RMSD values are extremely low. Although the docking power can still be well reflected even when the crystal poses are included and some popular benchmarks (e.g., CASF [58]) developed the datasets in this way, here we uniformly eliminated the crystal poses from the test sets, which might better mimic the real-world scenarios where all the poses are generated by a certain docking program.

As shown in Fig. 1D, E, in terms of AUROC and Rs, the ML-based models own absolute superiority over the classical methods. This can be majorly attributed to the training way of those MLSFs, which is executed by maximizing the overall classification capability to distinguish the correct poses from incorrect ones. However, as for the classical methods, the scores of the poses for a certain complex are often quite close while those of the poses for different complexes vary considerably, and therefore in some cases the score of the best pose for a certain complex is even lower than that of the worst pose for another complex. And it can also partially account for why the inter-statistics values for MLSFs are usually higher than the intra-statistics while those for classical SFs are often in the opposite. Among all the MLSFs, E3FP_XGB inevitably performs the worst, with its inter-AUROC (0.662) slightly higher than randomness but its intra-AUROC (0.536) close to randomness, suggesting that the model can consistently learn the differences between the pure ligand binding poses for different complexes but can hardly learn effective information from the intra-difference among a set of poses for a certain complex. According to the AUROC and Rs values, the inclusion of the classical Vina energy terms can surely improve the performance (e.g., the inter-AUROC values for NNscore_XGB and NNscore-Vina_XGB are 0.863 and 0.824, respectively, and those for ECIF_XGB and ECIF + Vina_XGB are 0.851 and 0.878, respectively), but the further incorporation of the docking pose ranks did not improve the predictions anymore (e.g., the inter-AUROC values for NNscore_XGB and NNscore + Rank_XGB are 0.863 and 0.864, respectively).

As for the success rate, a more important and intuitive statistics, although it shows a substantially similar trend to AUROC and Rs, some differences can still be observed. Among all the classical methods, Prime-MM/GBSA shows the best performance (SR_1_ = 0.690). Our MLSFs can hardly beat Prime-MM/GBSA unless the Vina energy terms are included, as shown by NNscore_XGB (SR_1_ = 0.715) and ECIF + Vina_XGB (SR_1_ = 0.730). Further inclusion of the docking pose ranks can only slightly improve the prediction, and finally ECIF + Vina + Rank_XGB illustrates the best docking power (SR_1_ = 0.736). Actually, the Vina energy terms or docking pose ranks may be regarded as a correction for the original Vina SF or pose ranking, thus not only ensuring the bottom line of performance but also having the chance to gain improvements through the interaction information learnt from the training data.

### Comparison based on the threefold clustered-cross validation of the re-docked poses

**Fig. 2:**
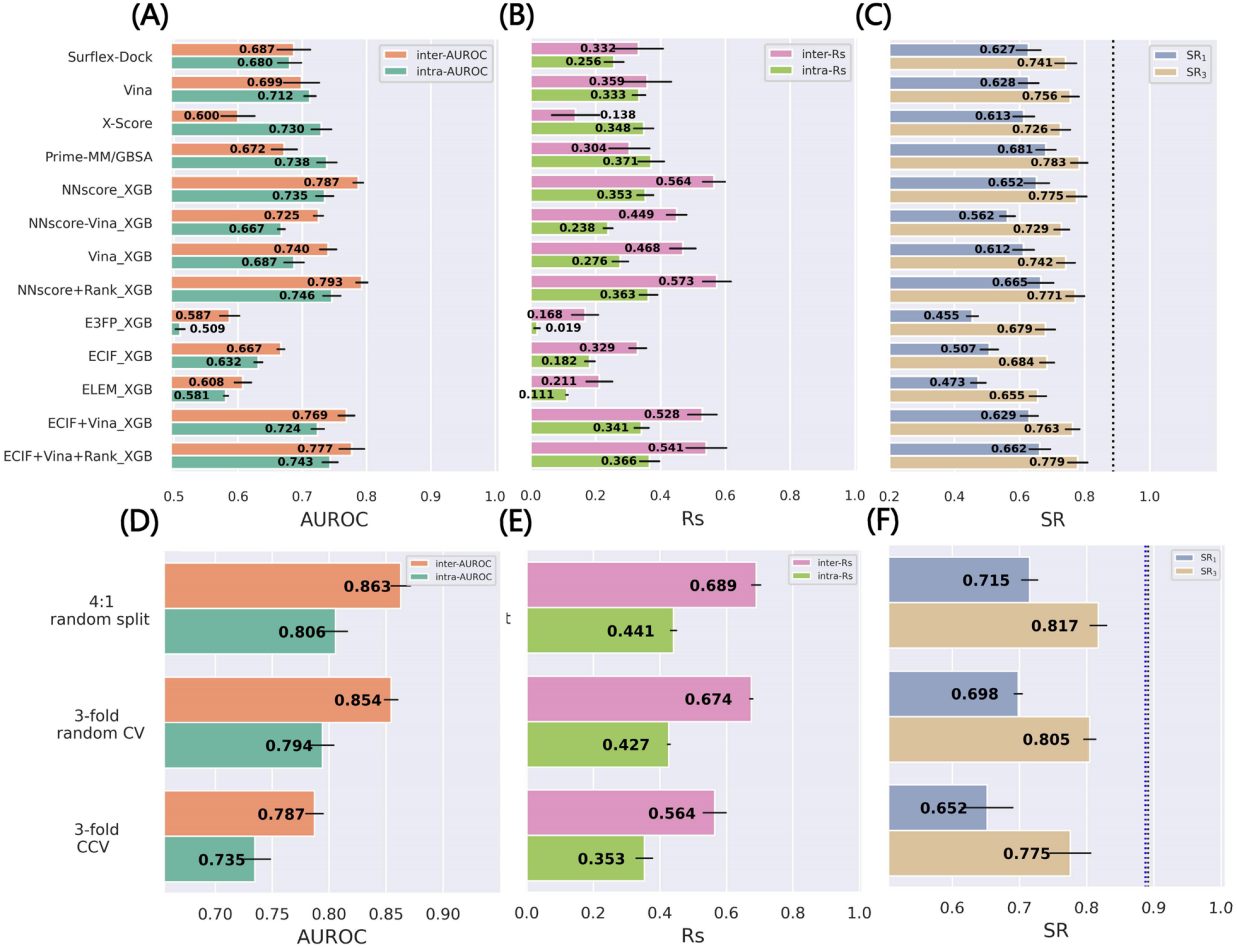
The performance in terms of **A** inter-AUROC and intra-AUROC, **B** inter-Rs and intra-Rs, and **C** top1 and top3 success rates based on the threefold clustered cross-validation (CCV) of the PDBbind-ReDocked dataset. A more intuitive comparison of different validation methods can be found in **D**–**F** taking NNscore_XGB as an example. The error bars represent the standard deviation of the 3 repetitions (10 repetitions for 4:1 random splitting). The dotted lines in **C** indicate the ceiling of the success rate, and those in **F** for 4:1 random splitting, threefold random CV, and threefold CCV are colored in black, purple, and blue, respectively

Compared with the results validated by the random splitting (Fig. 1), the MLSFs validated by the threefold CCV perform significantly worse but the performance of the classical methods is almost unchanged, as shown in Fig. 2A–C. To eliminate the influence of the training set size, we further conducted the threefold random CV, with the results for NNscore_XGB as an example depicted in Fig. 2D–F). The size of the training and test sets can surely affect the prediction accuracy (SR_1_ = 0.715 *vs* SR_1_ = 0.698), but it is obvious that removing similar samples for both proteins and ligands from the training set matters more (SR_1_ = 0.698 *vs* SR_1_ = 0.652). Under this circumstance, the best MLSF NNscore + Rank_XGB can only yield SR_1_ of 0.665, which is worse than Prime-MM/GBSA (SR_1_ = 0.681) but still better than other classical methods such as Vina (SR_1_ = 0.628). ML-based models have suffered from a long period of doubts for their poor generalization capability. A variety of studies have verified the impacts of the removal of the training proteins/ligands that are highly similar to the test proteins/ligands [30, 70–72], so here it may be not too surprising to see the performance decrease.

The performance decrease is especially prominent for the atomic pairwise counts-dominant methods, such as ECIF_XGB (SR_1_ = 0.686 *vs* 0.507), ELEM_XGB (SR_1_ = 0.559 *vs* 0.473) and NNscore-Vina_XGB (SR_1_ = 0.662 *vs* 0.562). ECIF_XGB and NNscore-Vina_XGB outperform Vina for the random splitting, but here their performance is remarkably poor, highlighting their sensitivity to the similar samples in the training set. Consistent with our previous study focusing on binding affinity prediction [30], the involvement of the Vina energy terms can alleviate this sensitivity, thus guaranteeing the bottom line of performance. Hence, it can also be expected to incorporate the energy terms from a more reliable classical SF rather than Vina in order to further improve the docking power and generalization capability of MLSFs.

### Comparison based on the refined-core splitting of the re-docked poses

**Fig. 3:**
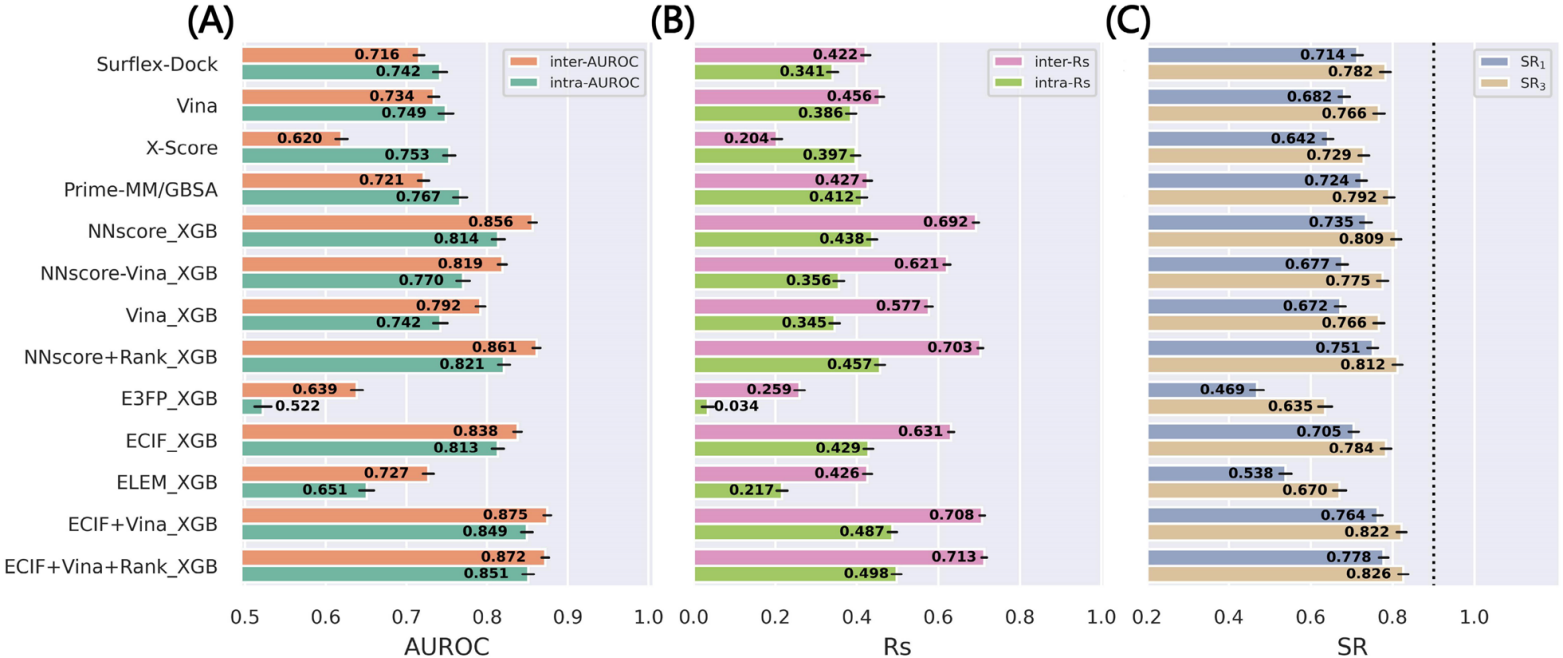
The performance in terms of **A** inter-AUROC and intra-AUROC, **B** inter-Rs and intra-Rs, and **C** top1 and top3 success rates based on the refined-core splitting of the PDBbind-ReDocked dataset. The error bars represent the standard deviation of the random sampling of 1000 redundant copies with replacements, and the dotted line in **C** indicates the ceiling of the success rate

The results for the refined-core splitting of PDBbind-ReDocked (Fig. 3) also illustrate a substantially similar trend to the above two validation methods, but there still exist several slight discrepancies especially for the methods based on NNscore and ECIF. Here, ECIF + Vina + Rank_XGB (SR_1_ = 0.778) has the best performance, followed by ECIF + Vina_XGB (SR_1_ = 0.764), NNscore + Rank_XGB (SR_1_ = 0.751) and NNscore_XGB (SR_1_ = 0.735), all of which rank higher than the classical Prime-MM/GBSA (SR_1_ = 0.724) and Surflex-Dock (SR_1_ = 0.714).

**Fig. 4:**
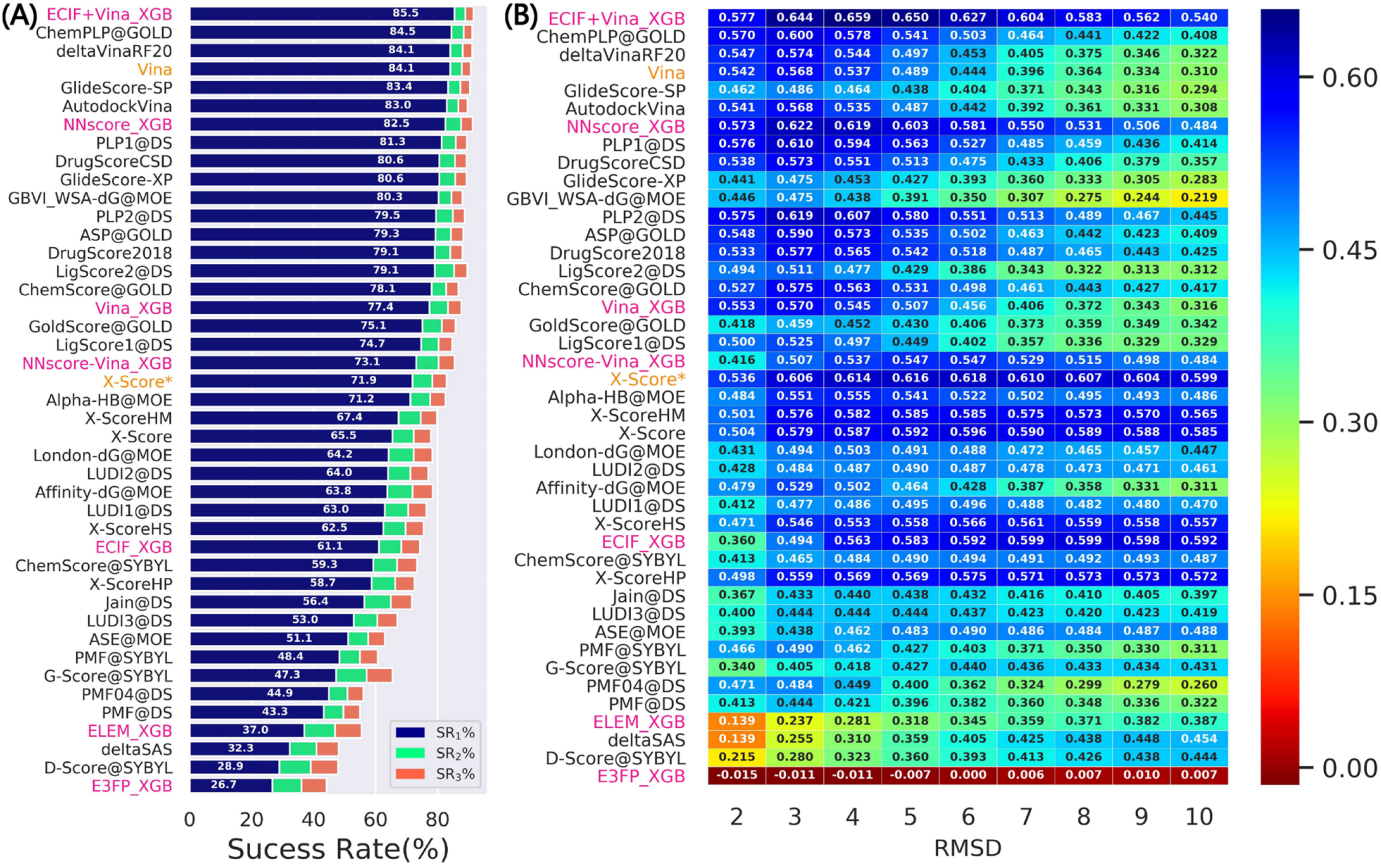
The performance in terms of **A** top1, top2 and top3 success rates, and **B** binding funnel analysis when the models are trained on the PDBbind-ReDocked-Refined set and tested on CASF-Docking set. The RMSDs utilized in the original CASF benchmark are employed here. The methods colored in pink and orange are calculated in this study, while the predicted scores of the others are just copied from original paper. The averages of the random sampling of 1000 redundant copies with replacements are shown in the figure. For binding funnel analysis, the x-axis indicates the RMSD range (e.g., [0–2 Å], [0–3 Å], etc.) where the Spearman correlation coefficients between the RMSD values and the predicted scores are calculated

To further assess the model performance and investigate whether the models can be generalized to the poses that own a wider distribution, we tested the models trained on the PDBbind-ReDocked-Refined set in CASF-Docking, mainly focusing on the success rates and binding funnel analysis utilized in the original CASF benchmark. As the RMSD values calculated in the original CASF benchmark and this study are not exactly the same, and hence the results based on these two sets of RMSDs are both presented (Fig. 4 and Additional file 1: Figure S2). The MLSFs that incorporate the docking pose ranks are not included because the binding poses in CASF were generated by three docking programs and thus the docking pose ranks can be hardly obtained. In addition, Prime-MM/GBSA is not tested here because the proteins/ligands should be prepared first to satisfy the requirement of Schrödinger, but here the calculations were conducted based on the proteins and ligand structures provided by CASF. Despite the lack of the docking pose ranks, ECIF + Vina_XGB can still rank the first in terms of the top1 success rate (SR_1_% = 85.5%). NNscore_XGB also has good performance (SR_1_% = 82.5%), but its rank is exceeded by some classical SFs such as Vina which do not show excellent performance discussed above. However, some ML-based models even rank in the latter places, such as ELEM_XGB (SR_1_% = 37.0%) and ECIF_XGB (SR_1_% = 61.1%). In fact, the authors of CASF have admitted that this set is an idealized case and the results reported here may be interpreted as the upper limit of the real performance. Anyway, this simple experiment can highlight the superiority of our best-performing MLSF and the importance of the inclusion of the Vina energy terms.

According to the binding funnel analysis shown in Fig. 4B and Additional file 1: Figure S1B, the superiority of the MLSFs seems more obvious. The aim of the binding funnel analysis is to estimate the rank correlation between the RMSD values and the predicted scores, which is similar to the Rs statistics described above. The only difference is that it further divides RMSD values into several windows, such as [0–2 Å], [0–3 Å], etc., to conduct a more comprehensive analysis. Compared with the top-ranked classical SFs in terms of the top1 success rate, both ECIF + Vina_XGB and NNscore_XGB do not show worse predictions and are significantly better if more high-RMSD poses are involved for analysis.

Another interesting finding is that the atomic pairwise counts-dominant MLSFs (such as ECIF_XGB and NNscore-Vina_XGB) tend to have better performance when more high-RMSD poses are included, suggesting their capability to recognize those extremely incorrect binding poses (e.g., the poses far from the binding pockets), while the energy term-centered methods (such as Vina_XGB and some of the classical SFs) have higher correlation coefficients among those low-RMSD poses, suggesting that they have better capability to rank high-quality binding poses. Moreover, the combination of these two types of features can result in more powerful classifiers, such as ECIF + Vina_XGB and NNscore_XGB.

### Training on re-docked poses and testing on cross-docked poses

**Fig. 5:**
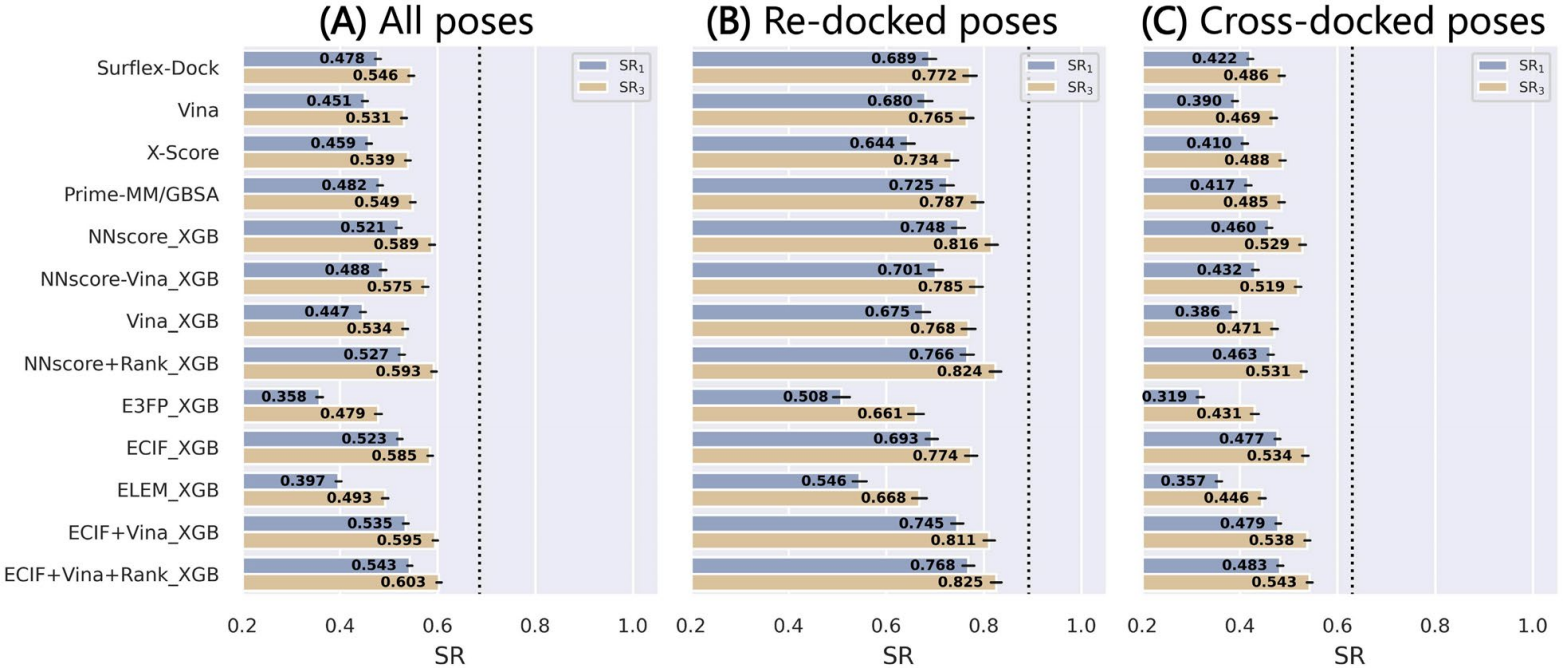
The top1 and top3 success rates of the models trained on PDBbind-ReDocked-Refined and tested on PDBbind-CrossDocked-Core-s, based on **A** all poses, **B** re-docked poses, and **C** cross-docked poses. The error bars represent the standard deviation of the random sampling of 1000 redundant copies with replacements, and the dotted line indicates the ceiling of the success rate

Protein flexibility is a particularly tough issue that impedes the applications of docking programs/SFs in SBVS [4, 5]. Most reported SFs were only assessed on the re-docked poses for their scoring/docking power, and thus in most cases they are over-estimated because some near-native poses cannot be well predicted due to the changeable residues and the possible steric conflicts in real-world scenarios. Hence, cross docking by docking the ligand of a certain target to the other crystal structures of the same target has emerged as an important method for the assessment of the docking power. The performance of the models trained on PDBbind-ReDocked-Refined and tested on PDBbind-CrossDocked-Core-s in terms of the success rate, AUROC, and Rs is illustrated in Fig. 5, Additional file 1: Figures S3 and S4, respectively. Compared with the corresponding performance based on the re-docked poses, the success rates and Rs values based on the cross-docked poses significantly decrease while the AUROC values change irregularly. This may be attributed to the fact that AUROC mainly reflects the overall classification capability of the models, so in the following the success rate will be majorly discussed. Despite remarkable decrease, we can still observe a substantially similar trend as the results based on the re-docked poses. Most MLSFs can still outperform the test classical SFs, among which Surflex-Dock in turn performs the best here (SR_1_ = 0.422). As for different featurization strategies for MLSFs, ECIF can yield an especially promising success rate, and the incorporation of the Vina energy terms and docking pose ranks can still achieve minor performance improvement (SR_1_ = 0.483 *vs* 0.479 *vs* 0.477 for ECIF + Vina + Rank_XGB, ECIF + Vina_XGB and ECIF_XGB, respectively). Even if the re-docked and cross-docked poses are combined to form a mixed set (Fig. 5A), we can still observe the superior performance of those best-performing MLSFs.

**Fig. 6:**
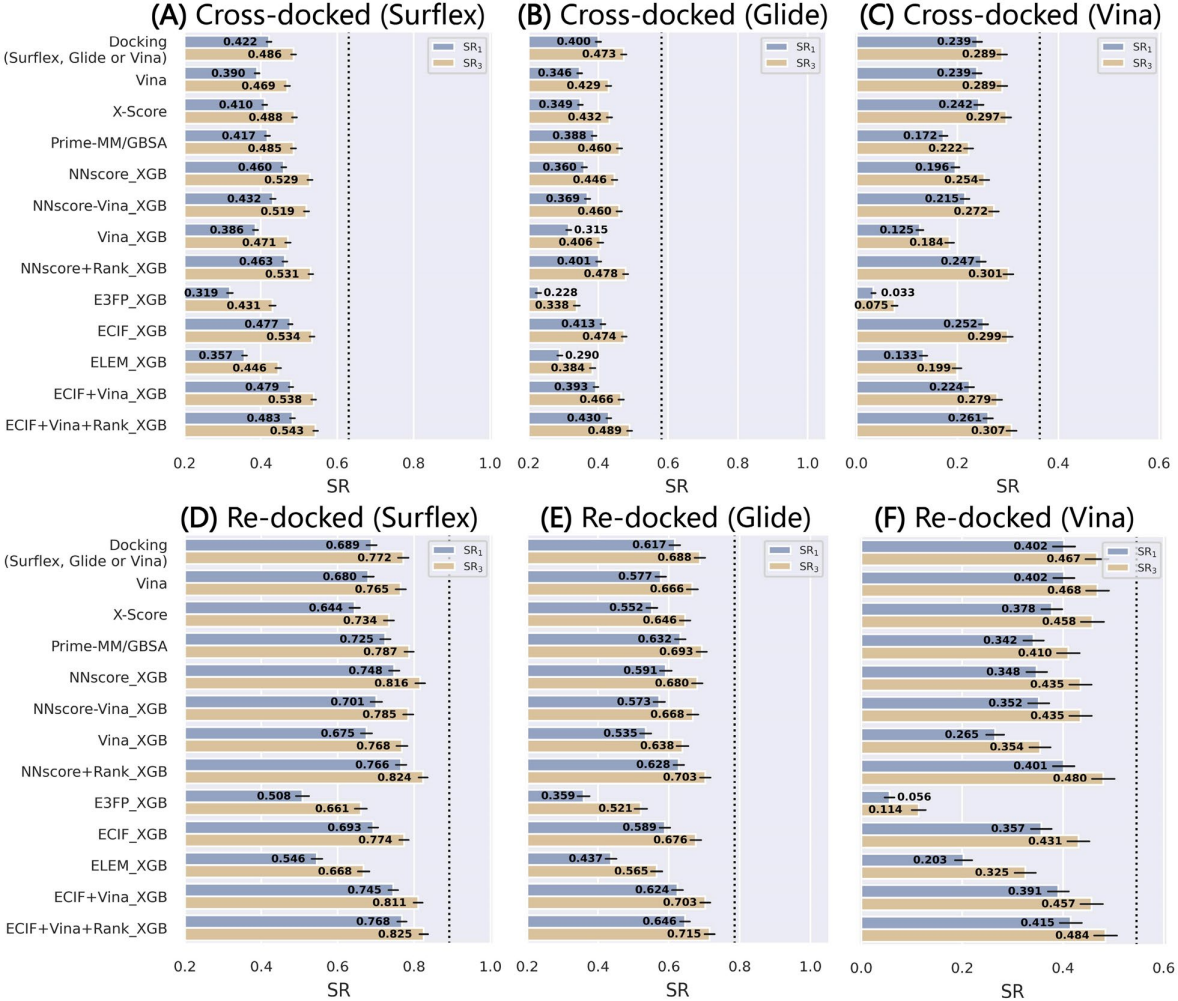
The top1 and top3 success rates of the models trained on PDBbind-ReDocked-Refined and tested on the **A** cross-docked and **D** re-docked poses in PDBbind-CrossDocked-Core-s, the **B** cross-docked and **E** re-docked poses in PDBbind-CrossDocked-Core-g, and the **C** cross-docked and **F** re-docked pose in PDBbind-CrossDocked-Core-v. The error bars represent the standard deviation of the random sampling of 1000 redundant copies with replacements, and the dotted line indicates the ceiling of the success rate

We then generalized the test sets to the other two datasets, PDBbind-CrossDocked-Core-g and PDBbind-CrossDocked-Core-v where the poses were generated by Glide SP and AutoDock Vina, respectively, to further investigate the sensitivity of these models to different docking programs. The performance in terms of the success rates can be found in Fig. 6, and those regarding the AUROC and Rs are depicted in Additional file 1: Figures S5 and S6, respectively, for reference. This experiment can to some extent verify the conclusion drawn from our previous study that the use of Surflex-Dock to generate the binding poses can increase the upper limit of the success rates [55], which are at least higher than those based on the binding poses generated by Glide SP and Vina. Overall, different methods perform differently on different sets, and most MLSFs cannot always beat the classical SFs. The only exception is the ECIF + Vina + Rank_XGB, which can yield acceptable predictions in all six cases based on either the re-docked or cross-docked poses. Another interesting finding is that the inclusion of the Vina energy terms here seems unfavorable to the predictions of the cross-docked poses generated by Glide SP (SR_1_ = 0.413 *vs* 0.393 for ECIF _XGB and ECIF + Vina_XGB, respectively) or Vina (SR_1_ = 0.252 *vs* 0.224), and is only slightly favorable to those of the cross-docked poses generated by Surflex-Dock (SR_1_ = 0.477 *vs* 0.479). A possible explanation may be that the protein–ligand interaction patterns for the re-docked and cross-docked poses are not exactly the same and hence the models can learn little from the pure re-docked poses to gain the information of the cross-docked poses.

Taken together, it seems that those ML-models trained on the re-docked poses can be well generalized to the re-docked or cross-docked poses generated by the same docking program. For the pose space defined by other docking programs, their performance is limited, especially for the predictions of cross-docked poses. Hence, a feasible strategy is to enlarge the training set, either through the augmentation and the diversification of the pose space for a certain complex or through the involvement of more complexes in the training set.

### Training on cross-docked poses and testing on re-docked/cross-docked poses

**Fig. 7:**
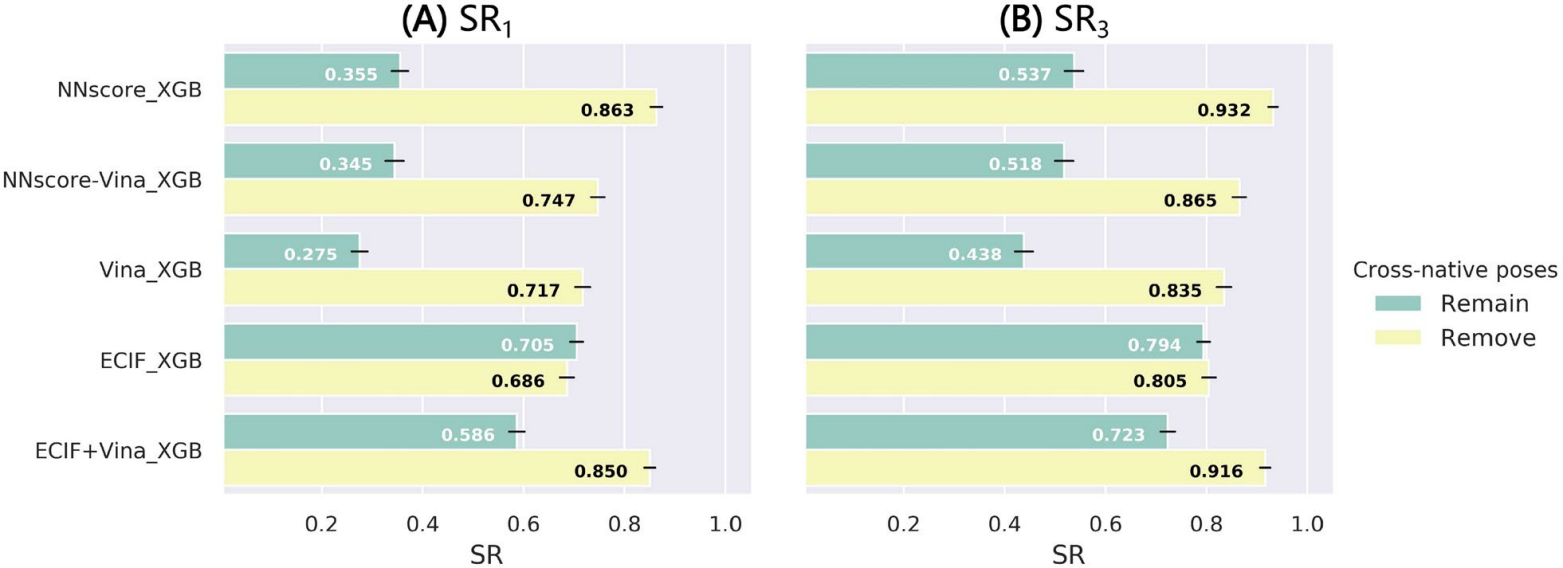
The impacts of the inclusion of the cross-native poses in training set on the top1 and top3 success rates of the models trained on PDBbind-CrossDocked-Refined and tested on CASF-docking. The error bars represent the standard deviation of the random sampling of 1000 redundant copies with replacements

To address the issue left in the previous section, we try to enlarge our training set by introducing the cross-docked poses into the training set, thus creating the PDBbind-CrossDocked-Refined set. At first, we also tried to include the native pose of each cross-docked complex (cross-native pose), which was generated through the alignment of two crystal structures regardless of the possible steric conflicts, in the training set just as we have conducted for the re-docked poses. Although they exert little effects on other test sets (e.g., PDBbind-ReDocked-Core), the performance on CASF-docking is awfully poor, as shown as Fig. 7. Except ECIF_XGB, the other models can gain prominent improvements when removing those native poses from the training set, suggesting that these models have learnt incorrect information from the cross-native poses. We guess that two reasons may majorly account for the higher sensitivity of this dataset to these incorrect cross-native poses. Firstly, the poses in CASF-docking were manually preprocessed and clustered, so that they are uniformly distributed in each RMSD window; secondly, CASF-docking owns more poses for a certain complex than the other test sets and even the training set (at most 100 *vs* at most 20). As for the minor influence on the ECIF features, we guess that this type of pure atomic pairwise counts-based features may be insusceptible to the possible conflicts between the protein and ligand because it only relies on the counts within the predefined distance, while the NNscore (containing several interaction-pairwise counts) and Vina (some physics-based energy terms) features are obviously not the case. Anyway, we will not include these cross-native poses in the following experiments, and we also do not recommend this type of poses to be involved if the researchers would like to carry out a similar study in the future.

**Fig. 8:**
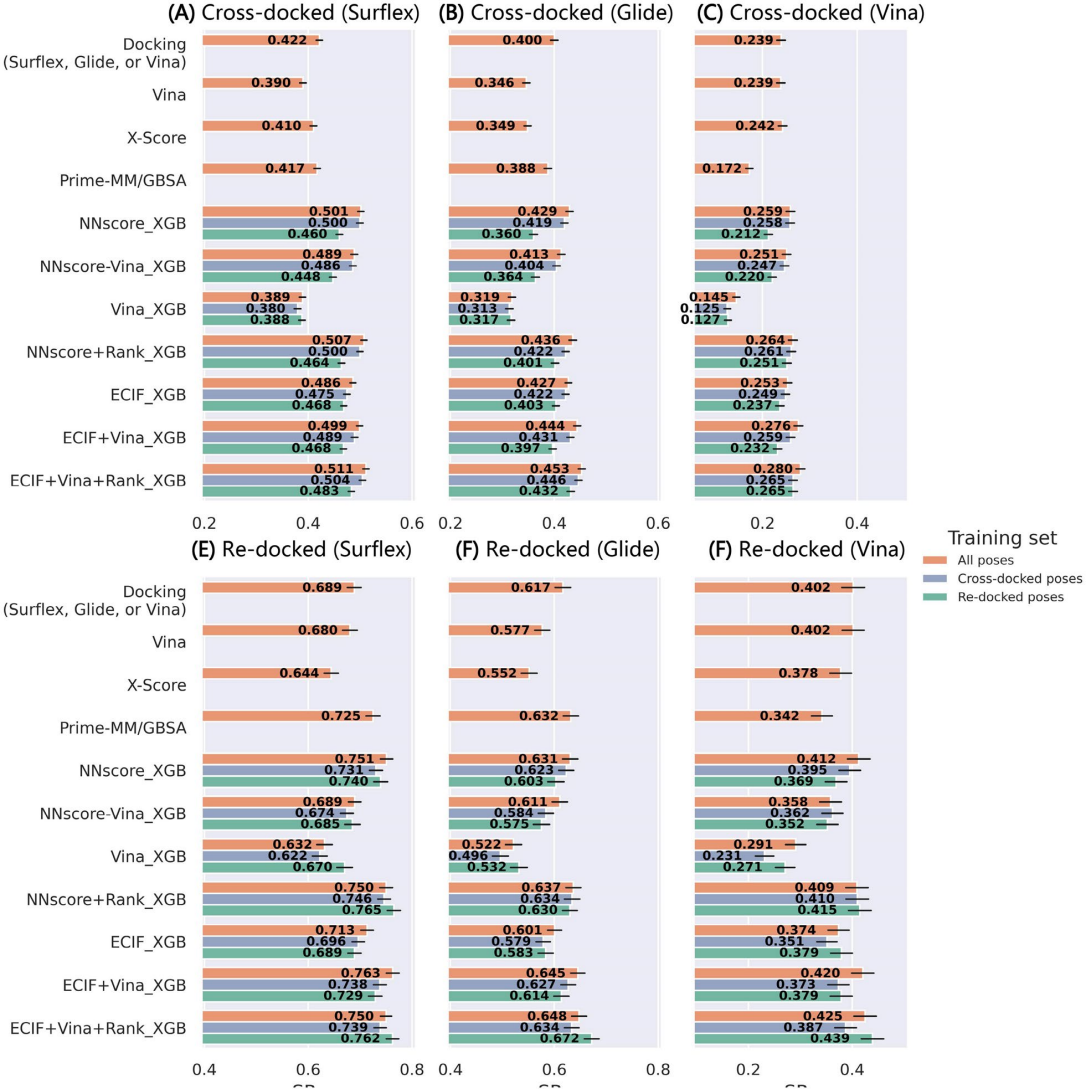
The impacts of the contents of the training set on the top1 success rates of the models trained on PDBbind-CrossDocked-Refined and tested on the **A** cross-docked and **D** re-docked poses in PDBbind-CrossDocked-Core-s, the **B** cross-docked and **E** re-docked poses in PDBbind-CrossDocked-Core-g, and the **C** cross-docked and **F** re-docked poses in PDBbind-CrossDocked-Core-v. The error bars represent the standard deviation of the random sampling of 1000 redundant copies with replacements

The performance of the models trained on the cross-docked poses and tested on the re-docked/cross-docked poses can be finally found in Fig. 8, and meanwhile, the sensitivity to the poses yielded by different docking programs and the impacts of the composition of the training set are systematically explored. We exclude E3FP and ELEM here to save computational costs, due to their poor performance before. The inclusion of the cross-docked poses in the training set can consistently improve the top 1 success rate for the cross-docked poses generated by whichever docking program, Surflex-Dock (Fig. 8A), Glide SP (Fig. 8B) or Vina (Fig. 8C), and the combination of the re-docked and cross-docked poses in the training set can further improve the performance for most MLSFs. However, the cases for the test sets only containing the re-docked poses are quite different (Fig. 8D–F). In some cases, the models trained on the re-docked poses can in turn yield the best docking power, especially for the methods that include the docking pose ranks as the features, such as NNscore + Rank_XGB and ECIF + Vina + Rank_XGB. The number of the cross-docked poses in the PDBbind-CrossDocked-Refined set is more than 20 times larger than that of the re-docked poses, but it seems that for some methods the expansion of the training set can hardly counteract the impact posed by the pose quality, suggesting that the disordered information learnt from the cross-docked poses is not necessarily favorable to pose prediction. Despite so, if we incline to develop MLSFs for real-world binding pose prediction, the inclusion of cross-docked poses in the training set is still necessary considering the complexity of protein–ligand interactions. As for different types of features, in most cases, the ECIF series show better performance than the NNscore series. With the addition of the cross-docked poses into the training set, the involvement of the Vina energy terms can finally improve the prediction accuracy in terms of the top 1 success rate, which is different from the results in the previous section. This finding further validates our previous conjecture that the poor performance of the Vina-included models may be contributed from the limited information learnt from the re-docked poses to predict cross-docked poses. The docking pose rank is surely an amazing feature for pose prediction, and its huge importance in performance improvement can be well recognized. Finally, compared with the classical baselines, the best model, ECIF + Vina + Rank_XGB, can surely exhibit its superiority in binding pose prediction, though it cannot always beat all the other MLSFs.

**Fig. 9:**
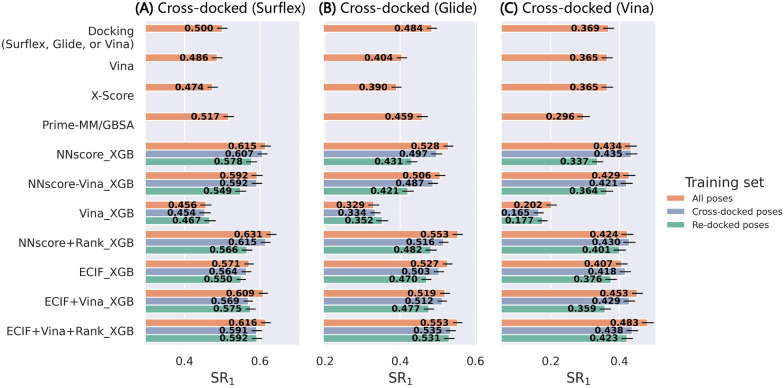
The top1 success rates of the models trained on PDBbind-CrossDocked-Refined and tested on the cross-docked poses in **A** in PDBbind-CrossDocked-Core-s, **B** PDBbind-CrossDocked-Core-g, and **C** PDBbind-CrossDocked-Core-v using the ensemble strategy. The error bars represent the standard deviation of the random sampling of 1000 redundant copies with replacements

We also report the results on the cross-docked poses when using the ensemble strategy (Fig. 9), where all the cross-docked poses of a certain ligand across multiple crystal structures are considered as a single set. From our point of view, the results depicted in Fig. 8 (A, B, C) can better mimic the real situation because we do not know which crystal structure is the best choice for a specific ligand, while our experiment just examines the potency of the ensemble docking strategy based on different rescoring methods. Compared with the original results, the ensemble strategy can indeed improve the performance, but compared with the predictions to the pure re-docked poses, the performance gap is still huge. This means that we can indeed improve the success rate for binding pose prediction through the combination of ensemble docking and rescoring, but they can hardly take the place of more ideal re-docked poses. As for other aspects such as the impact of the training sets, the impact of the featurization strategy, and comparison with classical methods, a substantially similar trend can be obtained as the results shown in Fig. 8, and hence we will not further discuss them here.

## Conclusions

Herein, several XGBoost-based classifiers designed for protein–ligand pose predictions were carefully validated through three rigorous validation methods. When both the training and test sets contain the re-docked poses, our MLSFs can surely exhibit superior performance to the classical methods, whichever based on the random splitting or refined-core splitting, or even tested on the dataset where the poses own a large coverage of RMSD distribution (i.e., CASF-docking). But as a common feature of the ML-based methods, the sequence/structural similarity of the proteins and ligands between the training and test sets consistently exerts a notable influence on the performance of MLSFs, which is reflected by a significant decrease of the performance of those methods when using the threefold CCV. However, although our best MLSF performs no better than Prime-MM/GBSA, it can still beat other commonly-used classical methods. Then, the models are also estimated with the involvement of the cross-docked poses in either the training or the test set. It seems that the ML models trained on the pure re-docked poses can only be well generalized to the re-docked/cross-docked poses produced by the same docking program used for the training set, but they cannot always outperform the classical methods when tested on the cross-docked poses generated by different docking programs. The incorporation of the cross-docked poses into the training set is favorable to enhance the performance on the cross-docked poses, but for the test sets with only the re-docked poses, the expansion of the training set by adding cross-docked poses sometimes can hardly counteract the influence of the pose quality. Besides the impacts of datasets, this study also demonstrates the importance of the inclusion of the classical energy terms or docking pose ranks as the features in binding pose prediction task, which can not only further improve the docking power to some extents but can ensure the generalization capability of the models.

Introducing ML technologies into SFs has emerged as a promising trend in recent years, but most relevant studies seem to pay more attention to binding affinity prediction or SBVS, rather than binding pose prediction, which has not been well achieved by traditional methods and has long been an important limiting factor for the further performance improvements of the former two tasks. As a supplement to the study conducted by Francoeur et al. [42], our study adopted a different way to handle the docking poses and employs a more direct way to validate the models. In addition, we further developed several pure PDBbind-based datasets, namely PDBbind-ReDocked (https://zenodo.org/record/5525936/files/PDBbind-CrossDocked-Core.tar.bz2), PDBbind-CrossDocked-Core (https://zenodo.org/record/5525936/files/PDBbind-CrossDocked-Core.tar.bz2), and PDBbind-CrossDocked-Refined (https://zenodo.org/record/5525936/files/PDBbind-CrossDocked-Refined.tar.bz2), for cross-docking experiments, which can be easily combined with the widely-used CASF benchmark/PDBbind dataset to conduct a more comprehensive assessment of SFs. Our study may provide sufficiently valuable guidance for the applications of MLSFs in binding pose prediction. Moreover, our datasets may serve as an important benchmark for further development and assessment of the MLSFs for protein–ligand binding pose prediction.

## Supplementary Information

**Additional file 1:** Additional Tables and Figures.

## Data Availability

The datasets, feature files, some representative scripts utilized in this study and an example to use the trained models to predict the binding poses of the in-house ligands can be available at https://github.com/sc8668/ml_pose_prediction with the MIT License and https://zenodo.org/record/5525936 with the Creative Commons Attribution 4.0 International license. The original source codes of NNscore, ECIF and E3FP can be available at https://git.durrantlab.pitt.edu/jdurrant/nnscore2, https://github.com/DIFACQUIM/ECIF and https://github.com/keiserlab/e3fp/tree/1.1, respectively. The source code of X-score can be available at https://www.ics.uci.edu/~dock/manuals/xscore_1.2_manual/.

## Abbreviations

CADD: Computer-aided drug design
SBDD: Structure-based drug design
SBVS: Structure-based virtual screening
3D: Three-dimensional
SF: Scoring function
ML: Machine learning
DL: Deep learning
CNN: Convolutional neural network
MLSF: Machine learning-based scoring function
ECIF: Extended Connectivity Interaction Features
E3FP: Extended three-dimensional fingerprint
ECFP: Extended connectivity fingerprint
RMSD: Root-mean-square-deviation
CASF: Comparative Assessment of Scoring Functions
CCV: Clustered-cross validation
XGBoost: Extreme gradient boosting
AUROC: Area under the receiver operating characteristic curve
Rs: Spearman’s rank correlation coefficient
SR: Success rate
