# Supplementary material for: The impact of cross-docked poses on performance of machine learning classifier for protein–ligand binding pose prediction
Source: J Cheminform. 2021 Oct 16;13:81. doi: 10.1186/s13321-021-00560-w (PMC8520186; doi:10.1186/s13321-021-00560-w)
Supplement: Supplementary file 1 — Additional file 1: Additional Tables and Figures. [file 13321_2021_560_MOESM1_ESM.docx]

**Additional file 1**

**The Impact of Cross-Docked Poses on Performance of Machine Learning Classifier for Protein-Ligand Binding Pose Prediction**

Chao Shen^1,2^, Xueping Hu^1^, Junbo Gao^1^, Xujun Zhang^1^, Haiyang Zhong^1^, Zhe Wang^1^, Lei Xu^4^, Yu Kang^1,*^, Dongsheng Cao^3,*^, Tingjun Hou^1,2,*^

^1^Innovation Institute for Artificial Intelligence in Medicine of Zhejiang University, College of Pharmaceutical Sciences, Zhejiang University, Hangzhou 310058, Zhejiang, P. R. China

^2^State Key Lab of CAD&CG, Zhejiang University, Hangzhou 310058, Zhejiang, P. R. China

^3^Xiangya School of Pharmaceutical Sciences, Central South University, Changsha 410013, Hunan, P. R. China

^4^Institute of Bioinformatics and Medical Engineering, School of Electrical and Information Engineering, Jiangsu University of Technology, Changzhou 213001, China

**Corresponding authors**

**Tingjun Hou**

**Tel:** +86-571-88208412

**E-mail**: tingjunhou@zju.edu.cn

**Dongsheng Cao**

**Tel:** +86-731-89824761

**E-mail:** oriental-cds@163.com

**Yu Kang**

**Tel:** +86-571-88208412

**E-mail**: [yukang@zju.edu.cn](mailto:yukang@zju.edu.cn)

**Table S1**. The tuned hyper-parameters in XGBoost.

| Hyper-parameters | Range |
| --- | --- |
| learning rate (*eta*) | [0.005, 0.5] |
| number of boosting stages (*n_estimators*) | [50, 3050] |
| maximum depth of a tree (*max_depth*) | [5, 20] |
| minimum loss reduction required to make a further partition on a leaf node of the tree (*gamma*) | [0, 1] |
| minimum sum of instance weight needed for a child (*min_child_weight*) | [1, 11] |
| subsample ratio of the training instances (*subsample*) | [0.5, 1] |
| subsample ratio of columns (*colsample_bytree*) | [0.5, 1] |
| subsample ratio of columns for each level (*colsample_bylevel*) | [0.5, 1] |
| subsample ratio of columns for each node (colsample_bynode) | [0.5, 1] |
| L2 regularization term on weights (*lambda*) | [0, 1] |
| L1 regularization term on weights (*alpha*) | [0, 1] |

**Table S2.** Comparison of the performance of machine learning classifiers and regressors based on the refined-core splitting of the PDBbind-ReDocked dataset. The standard deviations were calculated based on the random sampling of 1000 redundant copies with replacements.

| Method | Preditor | inter-AUROC | intra-AUROC | inter-Rs | intra-Rs | SR_1_ | SR_3_ |
| --- | --- | --- | --- | --- | --- | --- | --- |
| NNscore_  XGB | Classifier | 0.856 ± 0.005 | 0.814 ± 0.008 | 0.692 ± 0.007 | 0.438 ± 0.012 | **0.735** ± 0.014 | 0.809 ± 0.012 |
|  | Regressor | 0.845 ± 0.005 | 0.812 ± 0.008 | 0.695 ± 0.007 | 0.472 ± 0.012 | 0.733 ± 0.013 | 0.802 ± 0.012 |
| NNscore-Vina_  XGB | Classifier | 0.819 ± 0.006 | 0.770 ± 0.008 | 0.621 ± 0.008 | 0.356 ± 0.013 | **0.677** ± 0.014 | 0.775 ± 0.013 |
|  | Regressor | 0.802 ± 0.006 | 0.763 ± 0.009 | 0.616 ± 0.009 | 0.386 ± 0.012 | 0.666 ± 0.014 | 0.767 ± 0.013 |
| Vina_  XGB | Classifier | 0.792 ± 0.006 | 0.742 ± 0.009 | 0.577 ± 0.009 | 0.345 ± 0.013 | **0.672** ± 0.013 | 0.766 ± 0.013 |
|  | Regressor | 0.763 ± 0.006 | 0.707 ± 0.009 | 0.521 ± 0.010 | 0.279 ± 0.014 | 0.645 ± 0.015 | 0.752 ± 0.015 |
| NNscore+Rank_  XGB | Classifier | 0.861 ± 0.005 | 0.821 ± 0.007 | 0.703 ± 0.007 | 0.457 ± 0.012 | **0.751** ± 0.013 | 0.812 ± 0.012 |
|  | Regressor | 0.843 ± 0.005 | 0.812 ± 0.008 | 0.701 ± 0.007 | 0.462 ± 0.012 | 0.732 ± 0.014 | 0.809 ± 0.013 |
| E3FP_  XGB | Classifier | 0.639 ± 0.007 | 0.522 ± 0.011 | 0.259 ± 0.013 | 0.034 ± 0.015 | 0.469 ± 0.017 | 0.635 ± 0.017 |
|  | Regressor | 0.630 ± 0.007 | 0.525 ± 0.011 | 0.282 ± 0.012 | 0.053 ± 0.015 | **0.507** ± 0.017 | 0.659 ± 0.017 |
| ECIF_  XGB | Classifier | 0.838 ± 0.005 | 0.813 ± 0.008 | 0.631 ± 0.008 | 0.429 ± 0.012 | **0.705** ± 0.012 | 0.784 ± 0.013 |
|  | Regressor | 0.804 ± 0.006 | 0.762 ± 0.008 | 0.619 ± 0.009 | 0.394 ± 0.012 | 0.649 ± 0.013 | 0.749 ± 0.014 |
| ELEM_  XGB | Classifier | 0.727 ± 0.007 | 0.651 ± 0.009 | 0.426 ± 0.011 | 0.217 ± 0.013 | 0.538 ± 0.015 | 0.670 ± 0.016 |
|  | Regressor | 0.720 ± 0.007 | 0.656 ± 0.010 | 0.439 ± 0.012 | 0.240 ± 0.014 | **0.553** ± 0.016 | 0.691 ± 0.016 |
| ECIF+Vina_  XGB | Classifier | 0.875 ± 0.005 | 0.849 ± 0.007 | 0.708 ± 0.007 | 0.487 ± 0.011 | **0.764** ± 0.012 | 0.822 ± 0.012 |
|  | Regressor | 0.844 ± 0.005 | 0.815 ± 0.008 | 0.690 ± 0.008 | 0.455 ± 0.012 | 0.720 ± 0.013 | 0.793 ± 0.013 |
| ECIF+Vina+  Rank_XGB | Classifier | 0.872 ± 0.005 | 0.851 ± 0.007 | 0.713 ± 0.007 | 0.498 ± 0.012 | **0.778** ± 0.012 | 0.826 ± 0.012 |
|  | Regressor | 0.847 ± 0.005 | 0.829 ± 0.007 | 0.702 ± 0.007 | 0.463 ± 0.013 | 0.740 ± 0.013 | 0.811 ± 0.013 |


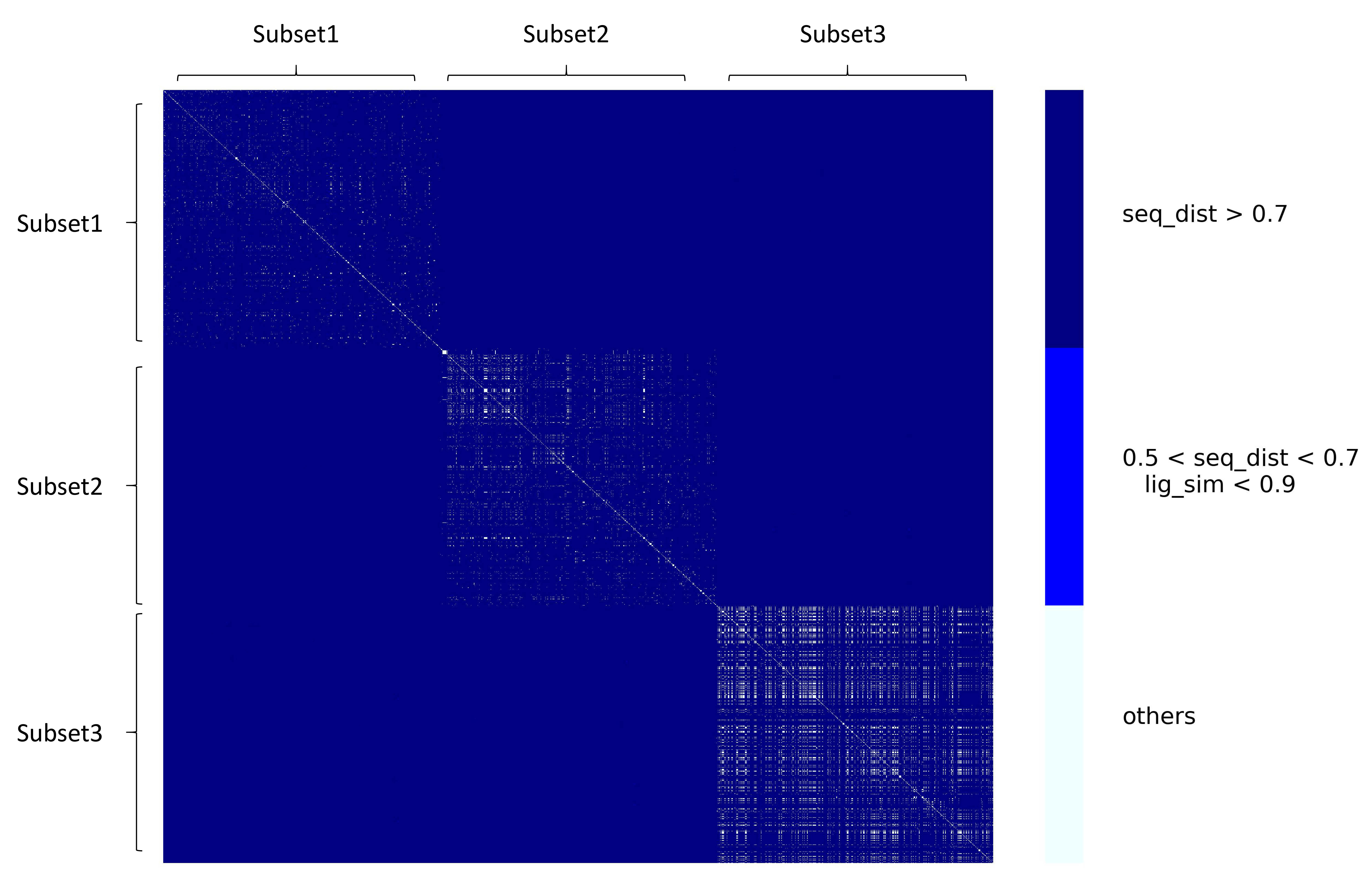


**Figure S1.** The similarity matrix between the different subsets when conducting 3-fold clustered cross-validation (CCV) of the PDBbind-ReDocked dataset. The *seq_dist* and *lig_sim* represent the sequence distance and ligand similarity between any two proteins/ligands, respectively.


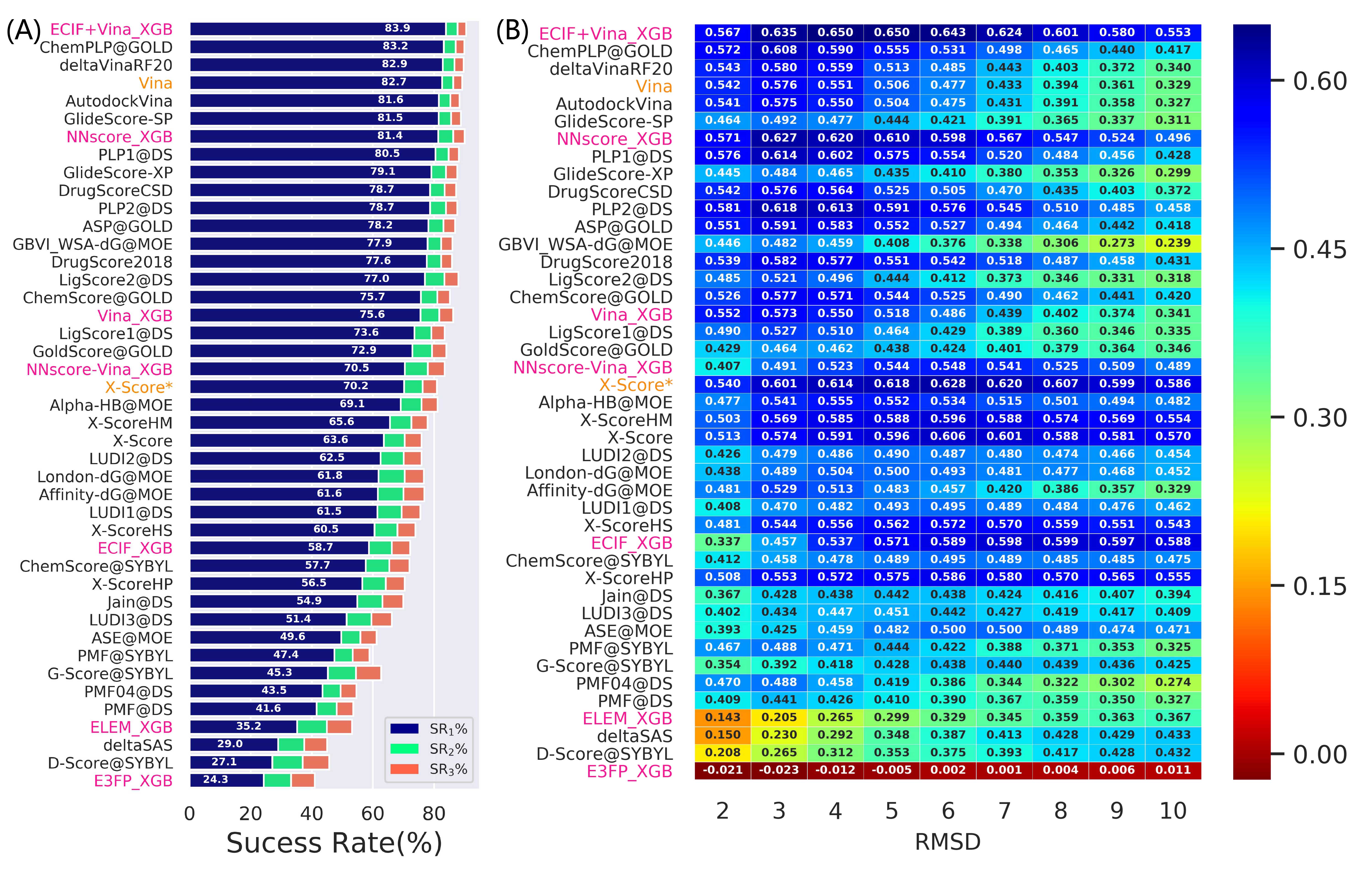


**Figure S2.** The performance in terms of (A) top1, top2 and top3 success rates, and (B) binding funnel analysis when the models are trained on the PDBbind-ReDocked-Refined set and tested on CASF-Docking set. The RMSDs are calculated using *obrms* utility in Openbabel. The methods colored in pink and orange are calculated in this study, while the predicted scores of the others are just copied from original paper. The averages of the random sampling of 1000 redundant copies with replacements are shown in the figure. For binding funnel analysis, the x-axis indicates the RMSD range (e.g., [0−2Å], [0−3 Å], and etc.) where the Spearman correlation coefficients between the RMSD values and the predicted scores are calculated.


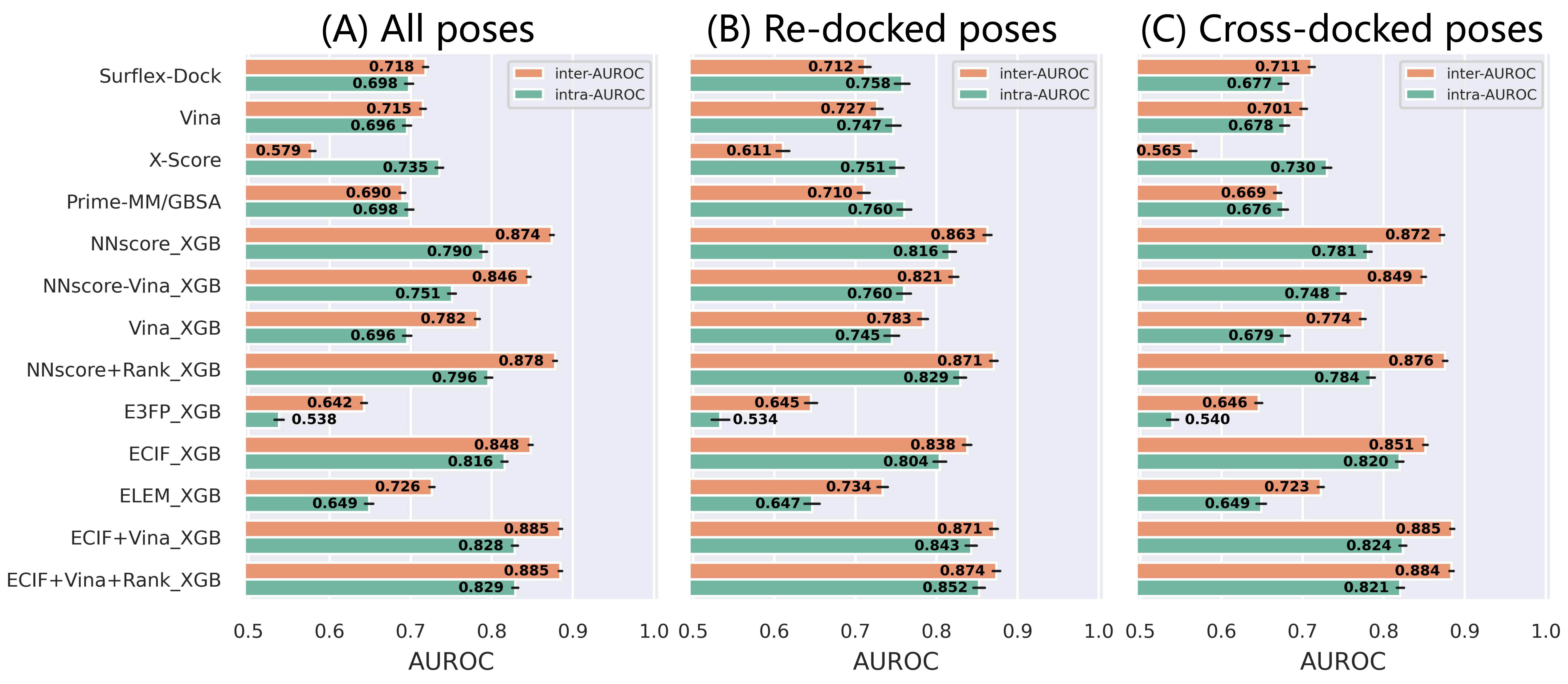


**Figure S3.** The AUROC values of the models trained on PDBbind-ReDocked-Refined and tested on PDBbind-CrossDocked-Core-s, based on (A) all poses, (B) re-docked poses, and (C) cross-docked poses. The error bars represent the standard deviation of the random sampling of 1000 redundant copies with replacements.


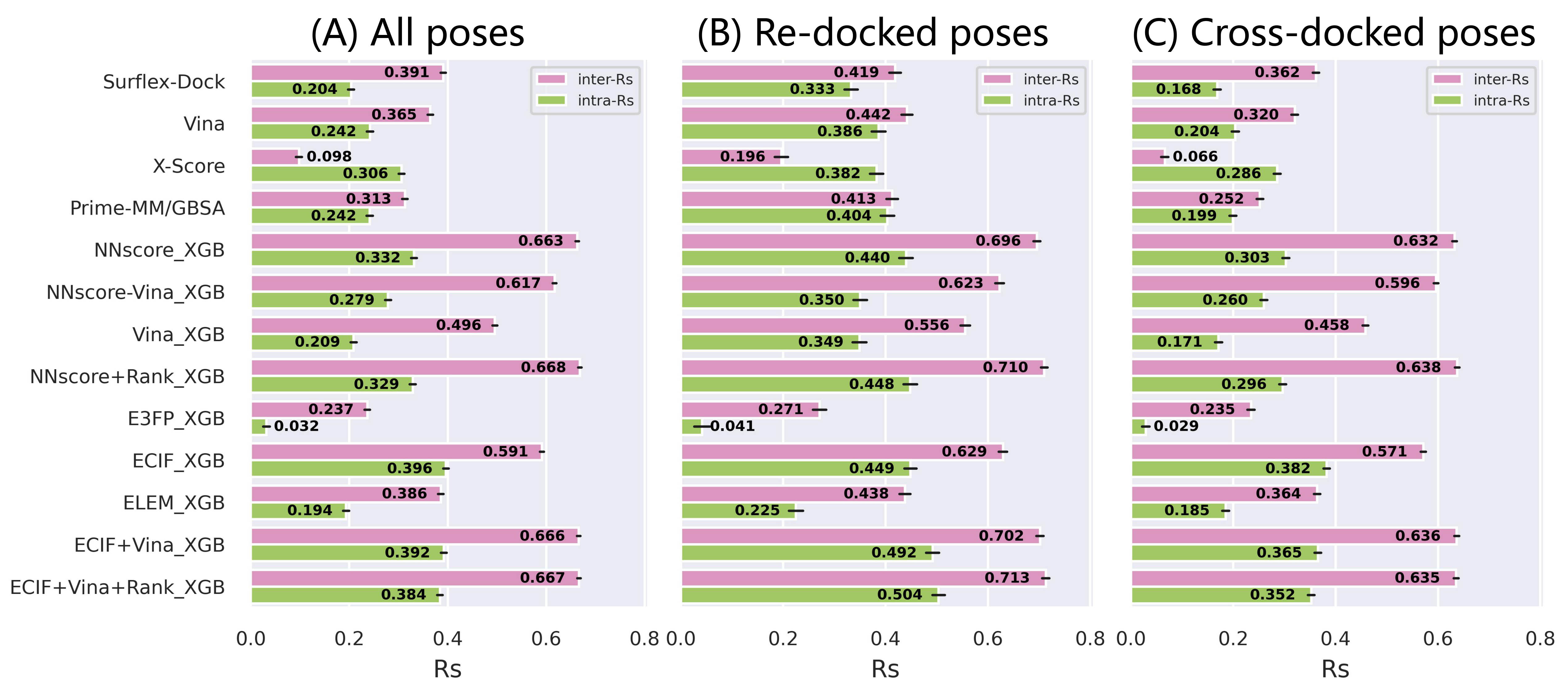


**Figure S4.** The Rs values of the models trained on PDBbind-ReDocked-Refined and tested on PDBbind-CrossDocked-Core-s, based on (A) all poses, (B) re-docked poses, and (C) cross-docked poses. The error bars represent the standard deviation of the random sampling of 1000 redundant copies with replacements.


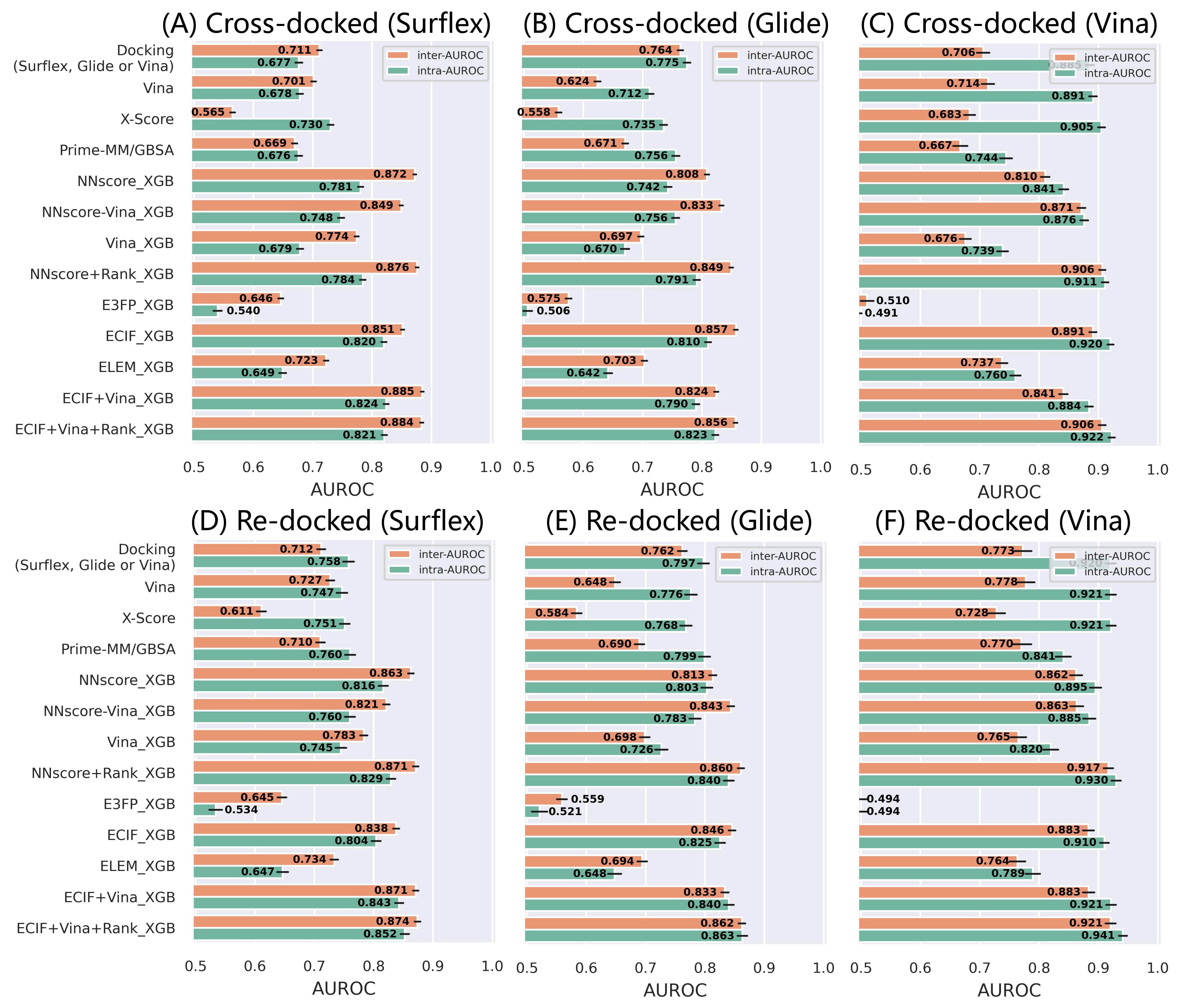


**Figure S5.** The AUROC values of the models trained on PDBbind-ReDocked-Refined and tested on the (A) cross-docked and (D) re-docked poses in PDBbind-CrossDocked-Core-s, the (B) cross-docked and (E) re-docked poses in PDBbind-CrossDocked-Core-g, and the (C) cross-docked and (F) re-docked pose in PDBbind-CrossDocked-Core-v. The error bars represent the standard deviation of the random sampling of 1000 redundant copies with replacements.


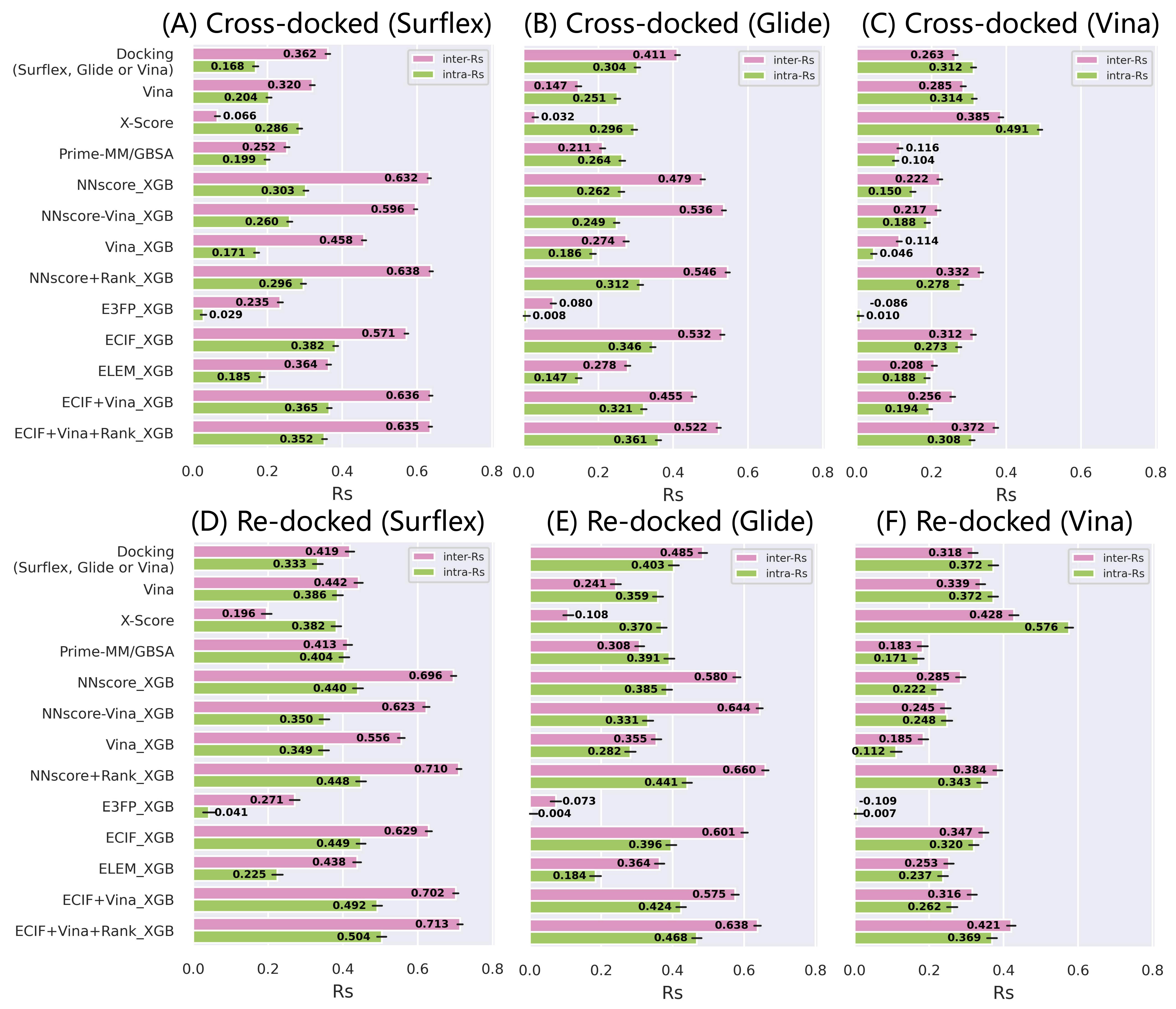


**Figure S6.** The Rs values of the models trained on PDBbind-ReDocked-Refined and tested on the (A) cross-docked and (D) re-docked poses in PDBbind-CrossDocked-Core-s, the (B) cross-docked and (E) re-docked poses in PDBbind-CrossDocked-Core-g, and the (C) cross-docked and (F) re-docked pose in PDBbind-CrossDocked-Core-v. The error bars represent the standard deviation of the random sampling of 1000 redundant copies with replacements.
